# Translational feasibility of a plasmonic microarray–based liquid biopsy for *KRAS* codon mutation detection across tissue, plasma, and urine in early colorectal cancer

**DOI:** 10.1038/s41698-026-01452-8

**Published:** 2026-05-02

**Authors:** Ji Young Lee, Chae Won Mun, Eun Ran Kim, Sung-Gyu Park, Min-Young Lee

**Affiliations:** 1 Advanced Bio and Healthcare Materials Research Division, Korea Institute of Materials Science (KIMS); 797, Changwon-daero, Seongsan-gu, Changwon-si, Gyeongsangnam-do, Republic of Korea; 2 Division of Gastroenterology, Department of Medicine, Samsung Medical Center, Sungkyunkwan University School of Medicine, Seoul, Korea

**Keywords:** Biological techniques, Biomarkers, Biotechnology, Cancer, Oncology

## Abstract

Accurate detection of KRAS codon mutations is essential for precision oncology in colorectal cancer (CRC), yet conventional liquid biopsy methods often lack sufficient sensitivity for rare ctDNA variants, particularly in early diseases. We developed a three-dimensional (3D) plasmonic KRAS microarray integrating blocked recombinase polymerase amplification with plasmon-enhanced fluorescence. Quencher-modified blocking probes suppress wild-type DNA while selectively enabling mutant signal amplification. A single primer–probe set per codon allows comprehensive detection of all substitutions within KRAS codons 12/13, 61, and 146. The platform achieved detection down to 1 fM by direct hybridization and 100 zM after blocked amplification, exceeding conventional PCR and next-generation sequencing sensitivity. Codon-level specificity was validated in CRC cell lines, with distinct signals for each mutation. Clinical analysis of 58 patients showed 100% concordance between tissue, plasma, and urine in mutation-positive malignant cases when sufficient input was available, indicating accurate reflection of tumor profiles. In benign tumors, detection was rare despite tissue mutations, likely due to limited ctDNA release.This plasmonic microarray enables ultra-sensitive, specific, and non-invasive detection, supporting early diagnosis, minimal residual disease monitoring, and longitudinal CRC management.

## Introduction

Although tissue biopsy remains the gold standard for cancer diagnosis and mutation analysis, it is inherently invasive and often limited by anatomical inaccessibility and procedural risks^1^. Furthermore, intratumoral heterogeneity may lead to sampling bias and incomplete molecular profiling^2^. With the increasing demand for precision oncology, liquid biopsy has emerged as a promising non-invasive alternative that enables the analysis of circulating tumor DNA (ctDNA) from body fluids such as blood and urine^3^. By capturing real-time molecular information, liquid biopsy addresses the limitations of spatial and temporal heterogeneity associated with tissue biopsies, and allows dynamic monitoring of treatment response, detection of minimal residual disease (MRDFi), and early identification of relapse^4^. As targeted therapies and immune checkpoint inhibitors become more prevalent in clinical practice, the need for reliable companion diagnostics has intensified^5^. However, detecting mutations in early-stage cancers remains technically challenging due to the extremely low abundance of ctDNA, necessitating highly sensitive and specific analytical platforms^6^.

Currently, the most widely used technologies for ctDNA analysis include PCR-based methods and next-generation sequencing (NGS)^7^. Quantitative PCR (qPCR) is widely used for its simplicity, cost-effectiveness, and speed in detecting known mutations. However, its performance declines in low-ctDNA settings due to limited sensitivity below 0.1–1% variant frequency^8^. This limitation stems from nonspecific amplification of wild-type sequences, which obscures rare mutant signals and reduces accuracy^9^. Additionally, its restricted multiplexing capability and susceptibility to false positives and negatives further constrain its clinical utility in early cancer detection or minimal residual disease monitoring^10^. Digital PCR (dPCR), which separates individual DNA molecules for analysis, significantly improves sensitivity (to as low as 0.01%) and quantification accuracy compared to qPCR^11^. However, its reliance on droplet or well partitioning imposes a physical limit on the number of measurable DNA molecules, which can restrict further sensitivity improvement^12^. Additionally, the high costs of equipment and reagents make it less suitable for large-scale screening^13^. NGS, capable of analyzing hundreds to thousands of gene mutations simultaneously, has advanced to include error-correction technologies such as Unique Molecular Identifiers (UMI) and duplex sequencing, allowing detection of mutations down to 0.01%^14^. Despite this, clinical applications still face challenges with background noise, a lack of standardization, long analysis times, and high costs^15^. Achieving consistent and reproducible detection of ctDNA in low-abundance settings, such as early-stage cancers, remains a major technical challenge due to limited tumor-derived DNA fragments and high background noise from normal cfDNA.

Currently, widely used liquid biopsy platforms include Guardant Health’s Guardant360®, Foundation Medicine’s FoundationOne® Liquid CDx, and Natera’s Signatera™. Guardant360® and FoundationOne® Liquid CDx perform NGS-based mutation analysis for dozens to hundreds of genes, providing valuable insights for patients who cannot undergo tissue biopsy^16^. However, their sensitivity is limited to around 0.1–0.5%, making them inadequate for detecting low-ctDNA concentrations and unsuitable for MRD detection^17^. In contrast, Signatera™ offers a highly sensitive approach by designing custom PCR primers based on tumor-specific mutations and performing NGS analysis, achieving sensitivities below 0.01%, making it ideal for MRD tracking^18^. However, the need for custom panel creation and analysis takes approximately five weeks, and its high cost and limited applicability in cases where tumor tissue is difficult to obtain pose challenges^19^. These commercial platforms are useful for advanced cancer patients, but they show limited sensitivity and specificity in early-stage cancer patients, where ctDNA concentrations are low^20^. Therefore, there is a pressing need for the development of new liquid biopsy technologies that offer affordable, rapid, and highly sensitive mutation detection, especially for early cancer stages.

To overcome these clinical and technical limitations, we developed an ultra-sensitive liquid biopsy platform that integrates plasmonic-based fluorescence signal amplification with suppression probe technology to inhibit wild-type nucleic acid amplification and background signal. Based on our previously reported success in detecting *EGFR* mutations in lung cancer patient blood samples^21^, this study expands to include not only blood but also urine cfDNA analysis in CRC patients, with a focus on optimizing both sensitivity and specificity for *KRAS* codon mutation detection. This technology was designed to enable the detection of all mutations within the target *KRAS* codon using a single primer–probe set, and its ultra-sensitive detection performance down to 10⁻⁹% was experimentally confirmed. Analysis of paired tissue, blood, and urine samples from 58 CRC patients (benign and malignant) revealed 100% concordance between tissue and cfDNA in both blood and urine for early-stage (stage 0–2) malignant CRC patients. Additionally, the clinical specificity was exceptionally high, over 90%. Compared to commercially available PCR and NGS platforms, this technology demonstrated superior sensitivity and accuracy, establishing its clinical utility.

## Results

### Strategy for detecting *KRAS* codon mutations in colorectal cancer patients

CRC is one of the most common and deadly cancers worldwide, and early detection, along with personalized treatment strategies, plays a crucial role in improving patient survival^22^. *KRAS* mutations serve as critical biomarkers for CRC, playing a pivotal role in diagnosis and treatment response prediction. *KRAS*, as a central component of the RAS/MAPK signaling pathway, regulates cell proliferation and survival, and its mutations are present in ~35–45% of CRC cases^23^. These mutations usually occur at specific codons—triplets of nucleotides in mRNA that code for individual amino acids. Single-nucleotide changes in these codons can alter the structure and function of the *KRAS* protein, leading to cancer development. In CRC, mutations are most commonly found at codons 12, 13, 61, and 146^24^. *KRAS* mutations at codons 12, 13, 61, and 146 are all associated with resistance to anti-*EGFR* therapies such as cetuximab and panitumumab^25–28^. Codon 12 is the most frequent site, accounting for ~70–80% of *KRAS* mutations in colorectal cancer, with several variants (e.g., G12V and G12D) also linked to poor prognosis^25^. Codon 13 represents the second most common site (~15–20%) and is likewise associated with resistance, though its clinical impact is generally less pronounced than codon 12^26^. Codon 61 mutations are less frequent (~1–4%) but can strongly activate RAS signaling and promote aggressive tumor behavior^27^. Codon 146 alterations occur in ~5–10% of cases and also drive constitutive pathway activation^28^. For early CRC screening, stool-based diagnostic tests have become widely used as a less invasive alternative to colonoscopy^29^. One well-known example is the FDA-approved Cologuard® test, which detects *KRAS* mutations, DNA methylation markers (such as NDRG4 and BMP3), and fecal occult blood simultaneously in stool samples^30^. This non-invasive, home-based collection method results in high patient compliance. However, stool-based testing is limited by the intermittent and inconsistent shedding of biomarkers, which is influenced by tumor location and intestinal transit time. In contrast, blood-based liquid biopsy enables the detection of ctDNA, which is continuously released from tumor cells and can provide prognostic information related to tumor burden, although it requires highly sensitive detection technologies.

In our previous work, we developed a highly sensitive detection platform for key mutations in the epidermal growth factor receptor (*EGFR*) gene—including exon 19 deletions, exon 20 insertions, and the exon 21 L858R point mutation. This platform combines a three-dimensional (3D) plasmonic substrate with a dual-probe strategy: fluorescence signal amplification is enhanced by common fluorescent probes that hybridize to both mutant and wild-type sequences on the plasmonic surface, while background suppression is achieved through quencher-labeled probes that selectively bind to the wild-type sequence. As a result, fluorescence signals are selectively amplified in the presence of mutant sequences at the target site, while wild-type sequences at the same site generate no signal. Using this approach, deletion and insertion mutations were detected with high specificity and accuracy. Even a single base insertion or deletion results in a significant sequence difference from the wild-type, which greatly reduces the likelihood of nonspecific probe hybridization. In contrast, point mutations—differing by only a single nucleotide—were identified using fluorescent probes precisely designed to hybridize with the mutant sequences, thereby ensuring both high analytical sensitivity and specificity. In this setup, a dedicated primer–probe set is required for the detection of each specific mutation.

On the other hand, codon-level mutations present a more complex challenge. Each codon—excluding the three stop codons (TAA, TAG, and TGA)—can theoretically mutate into any of the remaining 61 possible codon sequences. While it is technically feasible to design probes for all these variants, practical limitations such as cost and the restricted availability of clinical samples impose significant constraints on broad variant coverage. To address this limitation, we designed a 6-mer oligonucleotide probe (referred to as the wild-type inhibitor) labeled with a quencher, which selectively binds to the wild-type sequence at the target codon. This approach enables the detection of any mutation occurring within the codon region. For example, codons 12 and 13 of the *KRAS* gene, known hotspots for mutations, are located adjacent within the sequence; thus, a single quencher-labeled probe was designed to cover all potential mutations across these two codons within a six-nucleotide region. For other target codons that are spatially separated, individual 6-mer quencher-labeled probes were designed, incorporating adjacent sequences to increase binding specificity.

**Fig. 1:**
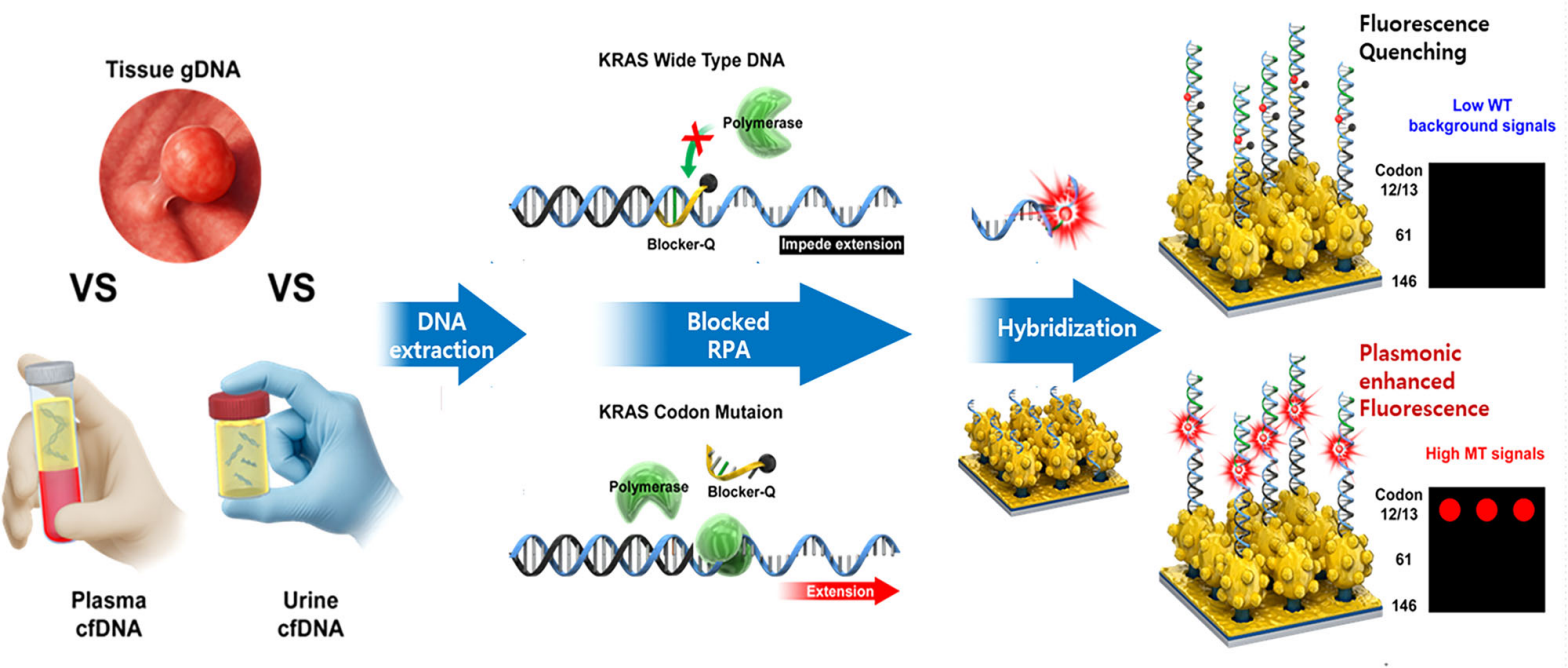
Schematic illustration of plasmonic microarray–based *KRAS* mutation detection from colorectal cancer patients. Circulating cell-free DNA extracted from plasma and urine is amplified through blocked recombinase polymerase amplification, in which wild-type *KRAS* sequences are suppressed by a quencher-modified probe while mutant codon sequences are selectively extended. The amplified products hybridize to probes on plasmonic nanostructures, producing low fluorescence background for wild-type sequences and plasmonic-enhanced fluorescence for mutant sequences, thereby enabling sensitive and specific detection.

As shown in Fig. 1, the overall analytical procedure consists of two main steps: nucleic acid amplification and hybridization onto a pre-fabricated three-dimensional (3D) nanoplasmonic microarray. Circulating cell-free DNA (cfDNA) extracted from patient samples was used to amplify a 100-nucleotide target region of the *KRAS* gene encompassing known mutation sites, using recombinase polymerase amplification (RPA) under isothermal conditions (39 °C for 30 min) in the presence of a wild-type-specific inhibitor. Primers were designed to anneal adjacent to the inhibitor binding site, thereby suppressing amplification of the wild-type sequence through competitive binding, while selectively amplifying mutant sequences to increase their relative proportion. After RPA, the phosphorylated reverse primer strand was digested with lambda exonuclease to generate single-stranded DNA (ssDNA). For fluorescence-based detection, Cy5-labeled probes complementary to sequences near the mutation sites—capable of binding to both codon mutant and wild-type sequences—and additional quencher-labeled wild-type inhibitors were mixed with the prepared single-stranded DNA (ssDNA) and applied onto a 3D plasmonic microarray where the probes had been pre-immobilized. In the presence of codon mutant sequences, the Cy5-labeled probes hybridized independently, enabling the plasmonic substrate to enhance the resulting fluorescence signals. In contrast, when wild-type sequences were present, both the Cy5-labeled probes and quencher-labeled wild-type inhibitors hybridized simultaneously, resulting in fluorescence quenching and inhibition of plasmonic enhancement, thereby minimizing background signals.

Figure S1 shows the fabricated 3D-nanoplasmonic substrate, consisting of uniform Au nanopillars densely decorated with Au nanoparticles across the surface. The resulting three-dimensional plasmonic substrate was sectioned into 10 × 10 mm squares for use in DNA microarray chip fabrication (Fig. S2). This detection platform was evaluated for its analytical sensitivity and specificity in detecting codon-level point mutations, and it was further validated using paired clinical samples—including tumor tissue, plasma, and urine—from patients with colorectal cancer, demonstrating its feasibility for clinical diagnostics. Additionally, it was compared and analyzed against existing commercial PCR and NGS technologies.

### Clinical characteristics of patients with malignant and benign colorectal tumors

A total of 58 patients were enrolled, including 39 with malignant colorectal tumors and 19 with benign colorectal tumors (Table 1). The mean age was 61.1 years (range, 34–80) for the malignant group and 63.4 years (range, 52–75) for the benign group, with sex distributions of 18 males and 21 females in the malignant group, and 9 males and 10 females in the benign group. The pathological stages were distributed as follows: stage 0 (*n* = 8), stage Ⅰ (*n* = 19), stage Ⅱ (*n* = 4), stage Ⅲ (*n* = 4), and stage Ⅳ (*n* = 4). Notably, 31 out of 39 malignant cases (79.5%) were collected at early stages (0–Ⅱ), indicating that our cohort predominantly consisted of early-stage colorectal cancers. Carcinoembryonic antigen (CEA) is a well-known tumor marker in colorectal cancer, but it is not recommended as a diagnostic marker because of its limited sensitivity and specificity. Although CEA levels were not available for all patients, among those with data, CEA elevation was observed in 10% (3/30) and 47% (14/30) of malignant cases when applying cut-off values of 5.0 and 2.5 ng/mL, respectively. In contrast, none of the benign cases exceeded the 5.0 ng/mL threshold, while 30% (3/10) exceeded the 2.5 ng/mL threshold. Consistent with these findings, our results also support that CEA has limited utility in early cancer detection. Detailed individual information is provided in Table S1.

**Table 1:** Clinicopathological characteristics

Tumor type	Pathologic stage	Patients number	Mean age (range)	Sex (M/F)	Histological type	CEA > cut-off value	
						>5.0 ng/mL	>2.5 ng/mL
**Malignant colorectal tumor (*n*** = **39)**	**Stage 0**	*n* = 8	61.08 (34–80)	18/21	Adenocarcinoma (*n* = 38) Neuroendocrine tumor (*n* = 1)	10% (3/30)	47% (14/30)
	**Stage Ⅰ**	*n* = 19					
	**Stage Ⅱ**	*n* = 4					
	**Stage Ⅲ**	*n* = 4					
	**Stage Ⅳ**	*n* = 4					
**Benign** **colorectal tumor (*n*** = **19)**	-	*n* = 19	63.42 (52–75)	9/10	-	0% (0/11)	30% (3/10)

Detailed individual data are provided in Table S1.

### Comparative analysis of KRAS mutations in tissue gDNA and plasma cfDNA using PCR-based commercial kits, with NGS performed on cfDNA

Tissue gDNA analysis was performed in all 58 colorectal tumor cases (39 malignant and 19 benign) using the PNAClamp™ *KRAS* Mutation Detection Kit (Ver. 4; Panagene, Daejeon, Korea). This assay is based on peptide nucleic acid (PNA)-mediated real-time PCR clamping, which selectively suppresses amplification of wild-type alleles to enrich mutant signals. The kit covers a total of 25 *KRAS* mutation targets, including 6 in codon 12, 6 in codon 13, 5 in codon 59, 12 in codon 61, 4 in codon 117, and 7 in codon 146. It has been approved by the Korean Ministry of Food and Drug Safety (MFDS) and carries the CE-IVD mark for tissue-based analysis, with a limit of detection (LOD) of ~2% mutant allele frequency. All mutation-positive tissue results identified by PNAClamp™ were further validated by Sanger sequencing, which was outsourced by the manufacturer to SolGent (Daejeon, Korea) as an independent sequencing provider. As shown in Table 2 and Table S2, *KRAS* mutations in tissue gDNA were identified in 31 of 58 colorectal tumor cases (53.4%), whereas the remaining 27 cases (46.6%) were mutation-negative. Among the malignant tumors, 18 of 39 cases (46.2%) were mutation-positive, with alterations most frequently detected at codon 12 (*n* = 14; p.G12V, *n* = 3; p.G12D, *n* = 9; p.G12A, *n* = 2), followed by codon 13 (*n* = 3; all p.G13D) and codon 146 (*n* = 2; p.A146T and p.A146V). One malignant case harbored dual mutations at codons 12 and 146, indicating multiple oncogenic alterations within a single tumor. In benign tumors, 13 of 19 cases (68.4%) were mutation-positive, including codons 12 (*n* = 7), 13 (*n* = 5), 61 (*n* = 1; p.Q61H), and 146 (*n* = 1; p.A146T). These findings suggest that oncogenic *KRAS* alterations can occur not only in malignant tumors but also in histologically benign lesions, emphasizing their potential importance for clinical monitoring and follow-up.

**Table 2:** *KRAS* mutation profiling in tissue and plasma using commercial *KRAS* detection kits

Tumor types	*KRAS* mutation status	Tissue gDNA by PNAClamp™ (LOD ~ 2%)		Sanger sequencing		Plasma cfDNA by PNAClamp™ (LOD ~ 2%)	Plasma cfDNA by ADPS™ (LOD ~ 0.1%)
**Malignant colorectal tumor (*n*** = **39)**	**Positive samples**	**Codon 12**	*n* = 14	p.G12V	*n* = 3	*n* = 0	*n* = 11
				p.G12D	n = 9		
				p.G12A	*n* = 2		
		**Codon 13**	*n* = 3	p.G13D	*n* = 3	*n* = 0	*n* = 0
		**Codon 61**	*n* = 0	**-**	**-**	*n* = 0	*n* = 0
		**Codon 146**	*n* = 2	p.A146T	*n* = 1	*n* = 0	*n* = 0
				p.A146V	*n* = 1	*n* = 0	n = 0
		**Total** ***n*** = **18**				**Sensitivity 0% (0/18)**	**Sensitivity 61% (11/18)**
	**Negative samples**	**Total** ***n*** = **21**				Not analyzed	**Specificity 52% (11/21)**
**Benign colorectal tumor (*n*** = **19)**	**Positive samples**	**Codon 12**	*n* = 7	p.G12V	*n* = 3	Not analyzed	Not analyzed
				p.G12D	*n* = 4		
				p.G12A	*n* = 0		
		**Codon 13**	*n* = 4	p.G13D	*n* = 4		
		**Codon 61**	*n* = 1	p.Q61H	*n* = 1		
		**Codon 146**	*n* = 1	p.A146T	*n* = 1		
		**Total** ***n*** = **13**					
	**Negative samples**	**Total** ***n*** = **6**					

Detailed individual data are provided in Table S2.

The *KRAS* mutation rate observed in the benign cohort (68.4%) was higher than that in the malignant cohort (46.2%). The benign lesions included in this study were primarily adenomas obtained through endoscopic resection. Notably, more than half of these cases (10/19, 52.6%) exhibited high-grade dysplasia, and a substantial proportion were tubulovillous adenomas containing a villous component, which are classified as advanced adenomas. According to the adenoma–carcinoma sequence model, *KRAS* mutation represents a key genetic event occurring at an early stage of colorectal tumorigenesis^31^. In particular, advanced adenomas with villous architecture or high-grade dysplasia have been reported to harbor *KRAS* mutations at relatively high frequencies^32^. These lesions are considered molecularly active pre-malignant stages characterized by the accumulation of genetic alterations. Importantly, even in the presence of high-grade dysplasia, lesions that do not invade beyond the basement membrane are not classified as invasive malignant carcinoma and remain pathologically diagnosed as adenomas. Therefore, the elevated *KRAS* mutation rate observed in the benign cohort likely reflects the pathological composition and molecular characteristics of the enrolled lesions rather than an unexpected biological phenomenon.

Based on the tissue gDNA mutation status, plasma cfDNA was comparatively analyzed using two commercially available *KRAS* detection kits, the PNAClamp™ *KRAS* Mutation Detection Kit and the ADPS™ *KRAS* Mutation Test Kit (GENECAST, Seoul, Korea) (Table 2). Firstly, plasma cfDNA analysis with PNAClamp™ was performed for the 18 malignant patients who were tissue *KRAS* mutation-positive; however, no *KRAS* mutations were detected, corresponding to 0% sensitivity. Plasma cfDNA from tissue *KRAS* mutation-negative malignant cases and benign tumors was not analyzed using PNAClamp™.

Plasma cfDNA was subsequently analyzed in malignant colorectal tumor patients using the ADPS™ *KRAS* Mutation Test Kit (GENECAST, Seoul, Korea), which targets 19 *KRAS* mutations located in exons 2 and 3, including codons 12, 13, and 61. The kit specifies the codon regions where mutations are interrogated but does not disclose the exact sequence-level information, such as the specific amino acid substitutions (e.g., G12D, G12V, and G12A). This assay employs a proprietary DNA polymerase with intrinsic discriminatory ability to directly recognize mutant alleles during amplification, enabling detection of low-abundance mutations with an analytical sensitivity of ~0.1%. The kit is currently designated for Research Use Only (RUO) and has not yet been approved for clinical diagnostic applications. Using ADPS™, 11 of the 18 tissue *KRAS* mutation-positive malignant cases (61% sensitivity) were detected as mutation-positive. In addition, misclassification was observed in two codon 13 mutation samples that were incorrectly identified as codon 12 (G12X). Moreover, false positives were reported in 11 of 21 tissue *KRAS* mutation-negative malignant cases, corresponding to a specificity of 47.6%. Plasma cfDNA from benign tumors was not analyzed using ADPS™. Detailed individual mutation profiles are provided in Table S2.

These findings demonstrate the challenges of plasma cfDNA–based *KRAS* mutation detection using current PCR-based commercial kits. The complete lack of detection by PNAClamp™ highlights its limited sensitivity for cfDNA analysis, likely due to the low abundance and fragmented nature of circulating tumor DNA. By contrast, ADPS™ showed substantially improved sensitivity (61%) owing to its discriminatory polymerase-based amplification, but its high false-positive rate raises concerns regarding assay specificity and clinical interpretability. Both assays rely on Ct value differences to distinguish mutant from wild-type alleles, yet nonspecific amplification of wild-type sequences can confound accurate discrimination. Sensitive assays operating near detection thresholds of ~0.1% may inadvertently amplify background noise or artifacts, thereby generating false-positive results. Collectively, these results suggest that currently available commercial kits provide only limited utility for plasma-based *KRAS* mutation detection. Further methodological refinement, rigorous validation with orthogonal confirmatory methods, and clinical contextualization are required to establish reliable and clinically actionable performance in cfDNA testing.

In addition, plasma samples (*n* = 18) from patients with tissue-confirmed *KRAS* mutations were analyzed using a Torrent-based electrical signal–driven liquid biopsy NGS service (GC-Genome); however, no *KRAS* mutations were detected. This result may be attributable to the analytical sensitivity and variant detection cutoff of the platform. Electrical signal–based NGS platforms generally achieve reliable detection at variant allele frequencies (VAFs) in the percent range, while low-frequency variants may fall below the limit of detection, resulting in false-negative findings. Considering that the majority of patients in this study were Stage 0 or Stage I, plasma ctDNA levels were likely extremely low. Under such conditions, the mutant allele frequency may fall below the detection threshold of conventional NGS-based assays, leading to non-detection.

### Analytical sensitivity of plasmonic *KRAS* microarray

Conventional solution-phase approaches for multiplex mutation detection, such as molecular beacons or TaqMan probes, face intrinsic limitations in both analytical sensitivity and specificity. Sensitivity is reduced because combining multiple probes in a single reaction elevates residual fluorescence background from incomplete quenching and probe instability. This elevated baseline lowers the signal-to-background ratio and increases the detection limit, making low-abundance mutant alleles more difficult to detect. Specificity is also compromised, as probe and primer competition frequently generate primer–dimer artifacts and uneven amplification efficiencies, leading to spurious signals and unreliable discrimination between wild-type and mutant sequences. From a multiplexing perspective, allele-specific PCR strategies, such as the Amplification Refractory Mutation System (ARMS), can provide high specificity for individual mutations but require separate primer sets for each variant, which becomes impractical for *KRAS* codons such as 12 and 13 that harbor multiple clinically relevant substitutions. This complexity raises cost and increases the likelihood of nonspecific amplification when scaled to multiplex detection. In contrast, the plasmonic *KRAS* microarray can improve both analytical sensitivity and specificity by separating amplification from detection and utilizing plasmonic-enhanced fluorescence (PEF). After blocking RPA, amplicons are hybridized to immobilized probes on a 3D plasmonic substrate, and wash steps remove unbound or excess fluorescent probes, thereby minimizing background and improving the signal-to-background ratio. Concurrently, PEF amplifies reporter emission through local electromagnetic field enhancement, enabling robust readout at lower fluorophore loadings and shorter exposure times, which can lower the limit of detection and reduce photobleaching. Spatial separation of capture probes across the array may further eliminate probe/primer competition in the readout stage and confine fluorescence to intended spots, supporting high specificity in multiplex. By combining single-set amplification, post-hybridization washing, and PEF, the microarray can achieve high analytical sensitivity and specificity and may provide a robust, scalable solution for multiplex *KRAS* mutation profiling in a clinically meaningful manner.

**Fig. 2:**
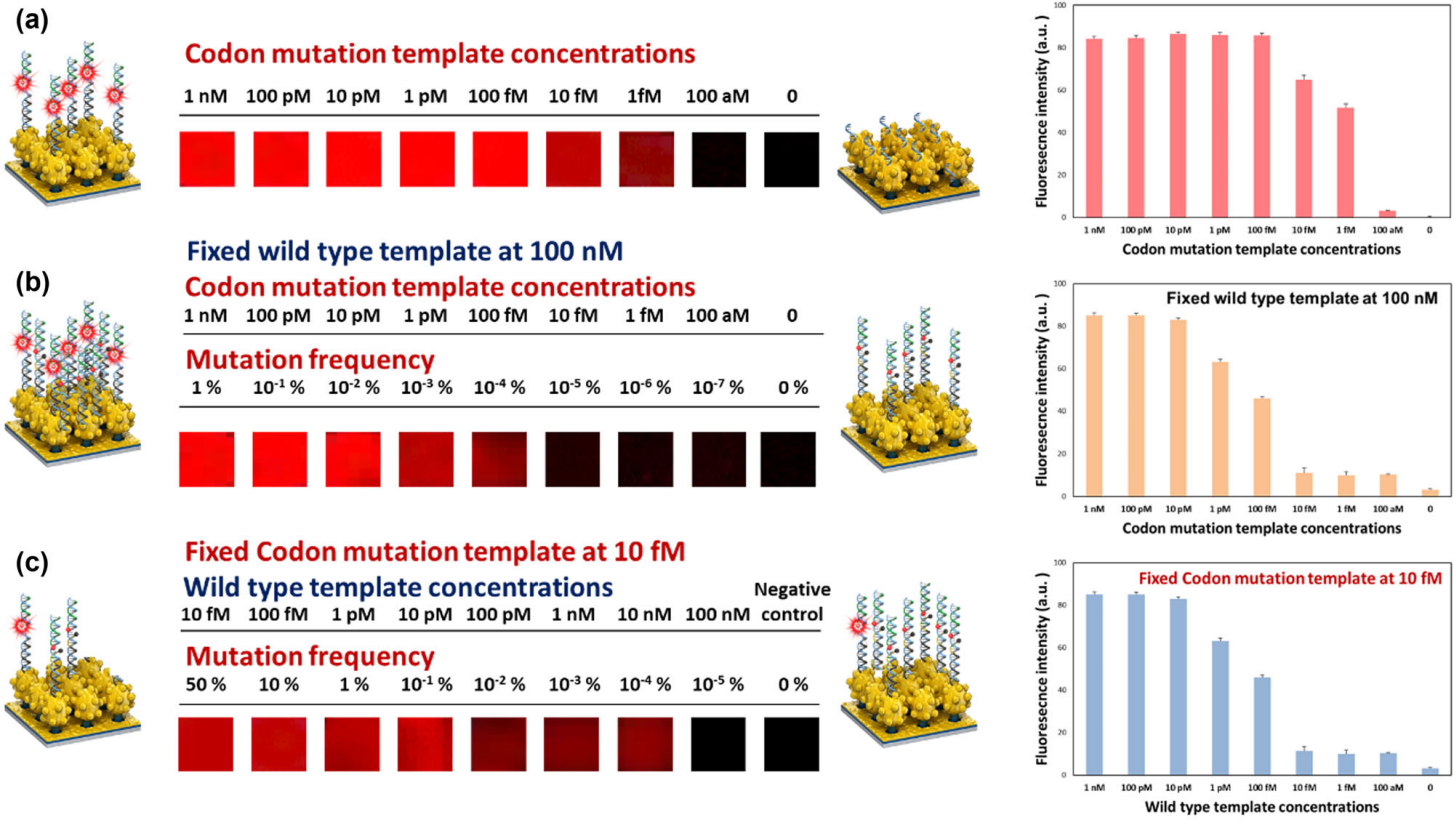
Analytical sensitivity assessment of the plasmonic substrate for codon mutation detection without DNA amplification. Fluorescence images of DNA hybridization without amplification: **a** varying codon 12 mutant DNA concentration, **b** fixed wild-type concentration with varying codon 12 mutant DNA concentrations, and **c** fixed codon mutant DNA concentration with varying wild-type concentrations.

Based on the *KRAS* mutation profiles obtained from colorectal tumor tissues, we selected clinically relevant hotspot codons 12/13, 61, and 146 as targets, and designed and synthesized the corresponding primer–probe sets. The microarray surface hybridization time was optimized by fixing the template concentration at 100 nM and evaluating incubation times of 10, 20, 30, and 40 minutes. As shown in Fig. S3a, fluorescence intensity reached a stable maximum at 30 min; therefore, 30 min was selected as the optimal hybridization time. To evaluate the analytical sensitivity of the plasmonic substrate for codon-specific mutation detection, we first examined direct hybridization of *KRAS* codon 12 mutant templates in the absence of wild-type DNA (Fig. 2a). Across a wide concentration range (1 nM–100 aM), fluorescence signals were consistently observed at high levels, indicating robust detection performance. Strong plasmon-enhanced fluorescence and efficient probe–target hybridization resulted in near-saturated signals across most concentrations. A gradual decrease in fluorescence was observed beginning at 10 fM, with a more noticeable reduction at 1 fM, likely due to the limited number of target molecules at extremely low concentrations, which reduces hybridization probability and introduces stochastic variation. Nevertheless, mutant DNA remained clearly detectable down to 1 fM, indicating a detection limit of 1 fM. To refine the quantitative region, four additional concentration points were introduced between 1 pM and 100 aM. Fluorescence intensity exhibited a linear relationship with the log₁₀-transformed target concentration over the range of 0.5 fM to 1 pM (*R*² = 0.9632), defining the linear dynamic range of the platform (Fig. S4).

In addition, the concentration of the wild-type blocker quencher probe was optimized under a fixed wild-type template concentration of 100 nM. Blocker concentrations of 5, 10, and 15μM were evaluated. At 10 μM, fluorescence derived from the wild-type template was effectively suppressed to a non-detectable level, while no further improvement was observed at 15 μM (Fig. S3b). Accordingly, 10 μM was selected as the optimal blocker concentration. Importantly, under this condition (10 μM blocker), mutant templates remained detectable down to 1 fM, confirming that selective wild-type suppression did not compromise mutant detection sensitivity (Fig. S3c). We next evaluated assay performance under conditions where wild-type and mutant DNA were mixed (Fig. 2b). Specifically, the wild-type template was fixed at 100 nM while the mutant concentration was varied. Fluorescence intensity decreased in proportion to the mutant DNA concentration, yet mutant signals were still detectable down to 100 fM (~10⁻⁴% frequency). These results demonstrate that the platform maintains high sensitivity despite a large excess of wild type. Wild-type DNA alone produced no measurable fluorescence signal because the quencher probe effectively suppressed background, confirming the specificity of the detection system. Finally, when the mutant template was fixed at 10 fM, and the wild-type concentration was increased from 10 fM to 100 nM (Fig. 2c), fluorescence intensity decreased in proportion to the wild-type background, reflecting competitive binding effects. Mutant signals, however, remained discernible up to 10 nM wild type (~10⁻⁴% frequency). Collectively, these results demonstrate that the plasmonic substrate enables codon-specific point mutation detection with high sensitivity and specificity, reaching a detection limit comparable to our previous reports on *EGFR* deletions, insertions, and point mutations. While conventional PCR and NGS typically discriminate mutation frequencies at the 0.1–1% level, this amplification-free plasmonic system distinguishes frequencies as low as 10⁻⁴%. This superior performance is attributed to plasmon-induced fluorescence enhancement and near-complete suppression of wild-type background by quencher probes, yielding a markedly improved signal-to-noise ratio and analytical sensitivity.

**Fig. 3:**
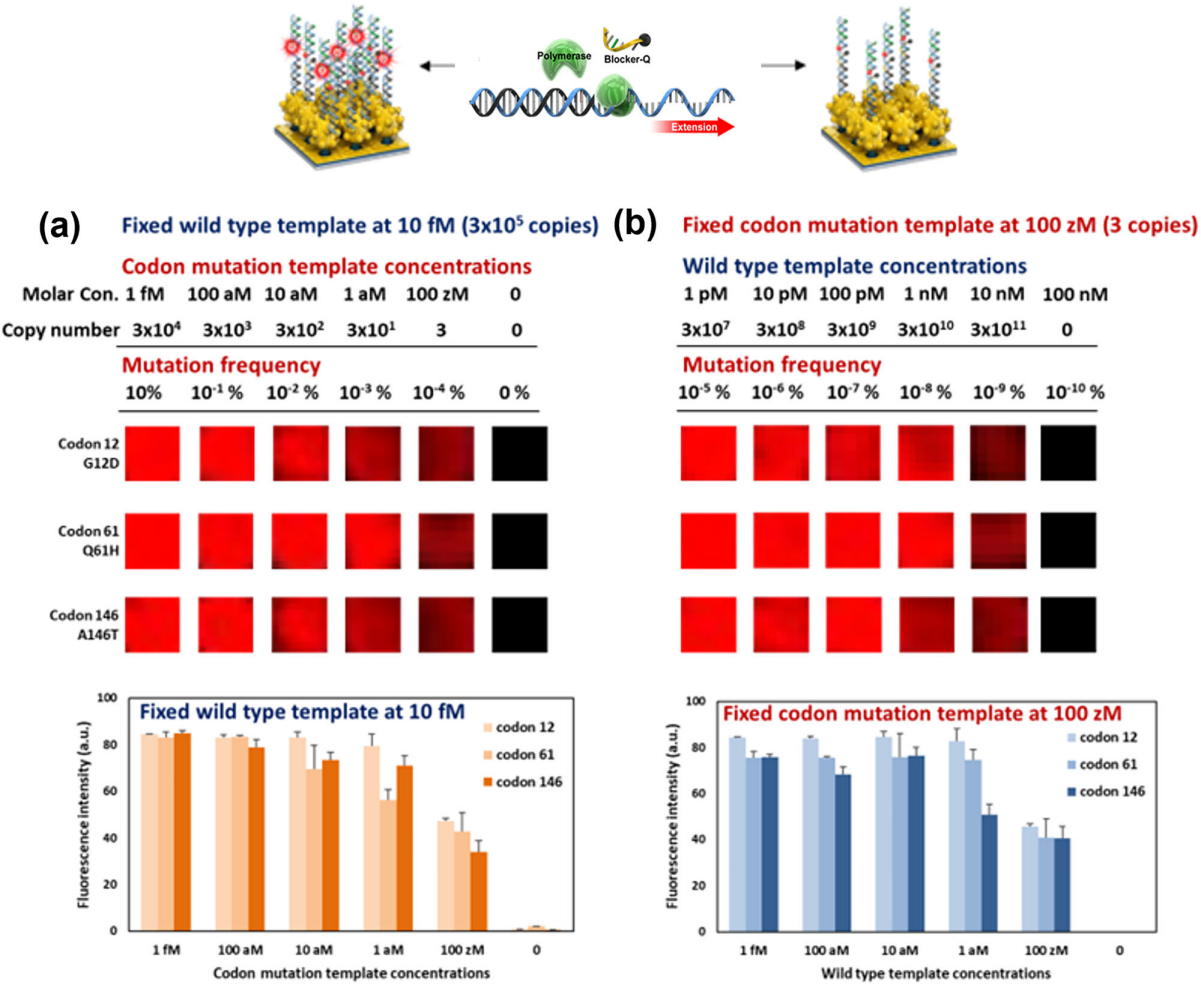
Analytical sensitivity assessment of the plasmonic substrate for codon mutation detection after blocked RPA amplification. Fluorescence images of DNA hybridization following blocked RPA amplification, shown separately for codons 12, 61, and 146: **a** fixed wild-type concentration with varying mutant DNA concentrations and **b** fixed mutant DNA concentration with varying wild-type concentrations.

To further evaluate the analytical sensitivity and selectivity, we performed blocked RPA amplification prior to hybridization on the plasmonic microarray under conditions where wild-type and codon mutant DNA were mixed. When the wild-type template was fixed at 10 fM and mutant DNA concentrations were varied from 1 fM (3 × 10⁴ copies/reaction) down to 100 zM (3 copies/reaction) (Fig. 3a), mutant-specific signals were clearly distinguished across the entire range, with reliable detection down to 100 zM (~10⁻⁴% frequency) for codons 12, 61, and 146. In contrast, no fluorescence signal was observed when only wild-type DNA was present, confirming the suppression of nonspecific background. Similarly, when the mutant template was fixed at 100 zM and the wild-type concentration was varied from 1 pM to 100 nM (representing mutation frequencies from ~10⁻⁵ to 10⁻¹⁰) (Fig. 3b), mutant-specific signals remained detectable up to a wild-type concentration of 10 nM (mutation frequency ~10⁻⁹). These results indicate that the blocked RPA step effectively suppressed nonspecific amplification from wild-type templates, enabling precise discrimination of mutant alleles at extremely low frequencies. Together, these findings confirm that the blocked RPA + hybridization on plasmonic microarray strategy markedly improves both the analytical sensitivity and specificity of mutation detection compared with direct hybridization alone.

To assess wild-type amplification suppression, SYBR Green real-time PCR was employed, as real-time kinetic analysis of RPA is technically limited (Fig. S5). The mutant template exhibited efficient amplification, whereas amplification of the wild-type template was markedly delayed in the presence of 10 μM quencher-labeled inhibitor. At 100 fM, the ΔCt between mutant and wild-type templates was 13.18 (~10⁻⁴-fold suppression), and at 10 fM, the ΔCt was 23.68 (>10⁻⁶-fold suppression). Suppression efficiency was therefore assessed based on Ct shifts rather than end-point fluorescence, as elevated final fluorescence at higher Ct values likely reflects late-cycle accumulation effects. These results confirm that the wild-type inhibitor substantially suppresses amplification of the wild-type template, thereby enhancing mutant detectability in a wild-type background.

At ultra-low mutation frequencies, stochastic sampling effects must be considered due to Poisson distribution–governed molecular occupancy. Under a reaction volume of 50 µL, 100 zM corresponds to ~1 × 10⁻⁹ % mutation frequency relative to 10 nM WT and yields an average molecular occupancy (λ) of ~3 copies per reaction. In this regime, the theoretical probability of at least one target molecule per reaction is ~95%, indicating that stochastic variation may influence detection outcomes. To ensure statistical robustness, additional experiments were conducted at 500 zM (≈5 × 10⁻⁹ % relative to 10 nM WT), corresponding to λ ≈ 15 copies per reaction, where zero-copy events become statistically negligible. Under these conditions, consistent fluorescence signals were observed across independent replicates (3/3), whereas no signal was detected in WT-only controls (0/3). These results support that ultra-low mutation detection is not attributable to stochastic artifacts or nonspecific background, but reflects reproducible detection under sufficient molecular occupancy conditions (Fig. S6).

To further examine assay performance under biologically relevant matrix conditions, synthetic mutant single-stranded DNA (100 mer) was spiked into fetal bovine serum (FBS) and commercially purchased healthy human urine, followed by cfDNA extraction and analysis using the same blocked RPA–plasmonic microarray workflow. Under these conditions, reproducible detection was achieved down to 10 fM in both matrices, representing a reduced sensitivity compared with the 100 zM limit of detection obtained under buffer-based conditions. This decrease in sensitivity is primarily attributed to target loss during the pre-analytical extraction process. In particular, at ultra-low input levels, even partial recovery loss can elevate the practical limit of detection. Nevertheless, mutant-specific signals remained clearly distinguishable from background in both matrices (Fig. S7).

We evaluated the stability of streptavidin–biotin functionalized microarrays after storage at 4 °C for one week. Sensitivity and signal-to-noise ratio (SNR) were maintained down to 1 fM at levels comparable to the initial performance (Fig. S8), indicating practical short-term stability under the specified storage conditions.

### Analytical specificity of plasmonic *KRAS* microarray

**Fig. 4:**
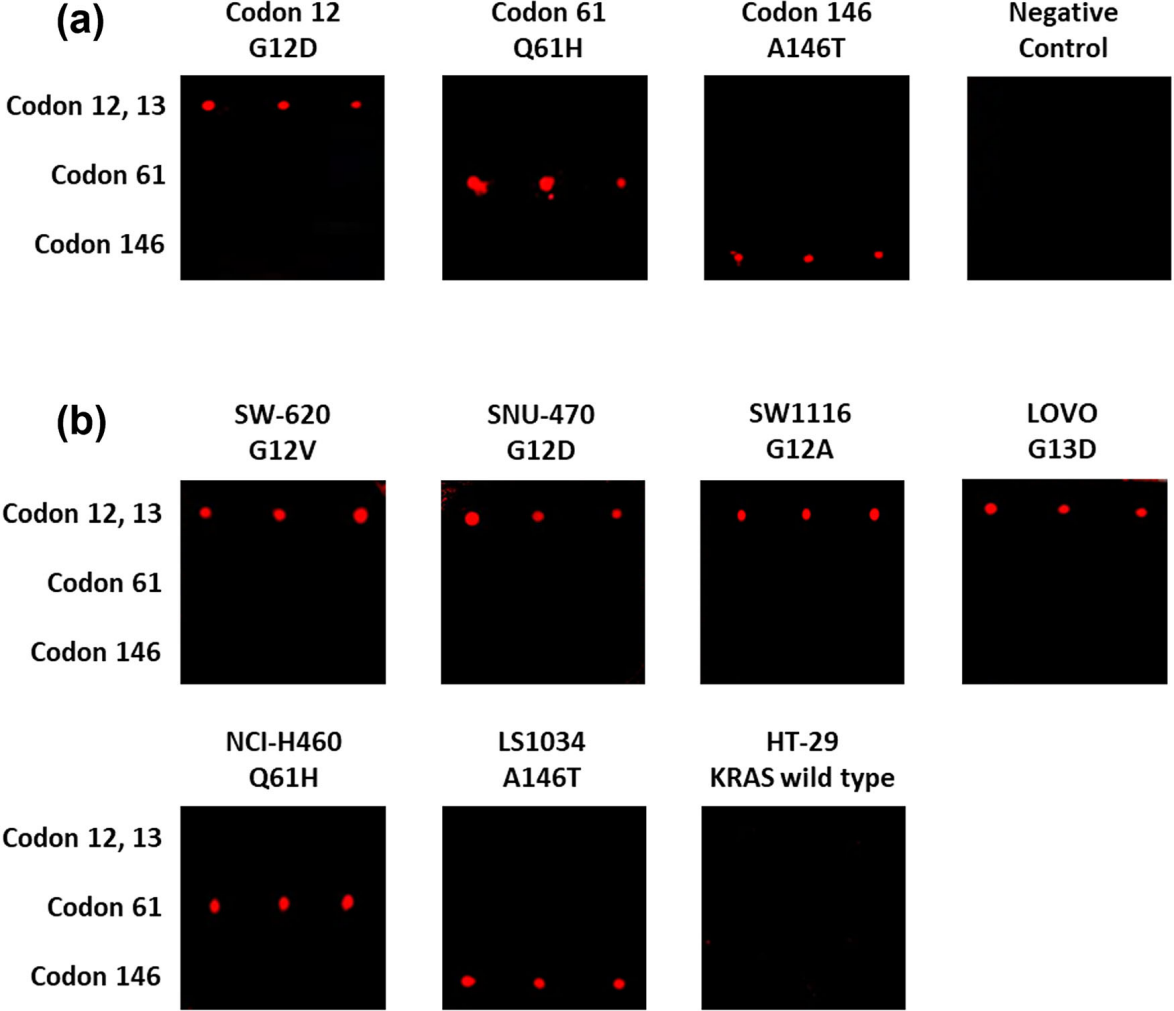
Analytical specificity assessment of the plasmonic *KRAS* microarray following blocked RPA amplification and hybridization. **a** Fluorescence images of the plasmonic *KRAS* microarray using synthetic DNA templates. **b** Fluorescence images of the plasmonic *KRAS* microarray using genomic DNA extracted from colorectal cancer cell lines. Quantitative results are provided in Fig. S2.

For multiplex *KRAS* mutation detection, a 3D plasmonic *KRAS* microarray was fabricated to allow simultaneous capture and analysis of amplicons corresponding to the clinically relevant hotspot codons 12/13, 61, and 146. Each biotinylated probe listed in Table 3 was premixed with streptavidin in a tenfold molar excess and spotted in 50 nL volumes onto 10 × 10 mm plasmonic substrates in a 3 × 3 array format. This fabrication strategy enabled parallel interrogation of multiple *KRAS* variants within a single microarray. For assay validation, synthetic target templates were generated according to the sequences listed in Table 3. Primers were designed to amplify genomic regions encompassing codons 12/13, 61, and 146, while quencher-labeled oligonucleotide probes complementary to the wild-type sequences were employed to suppress amplification of non-mutated alleles. Multiplex blocked RPA was then performed, and the resulting amplicons were hybridized onto the fabricated plasmonic *KRAS* microarray. Importantly, wild-type templates produced no fluorescence signals at any array spot, whereas mutant templates generated clear and spatially resolved signals only at their respective capture sites, confirming the high specificity of the assay (Fig. 4a). These results demonstrate that the 3D plasmonic *KRAS* microarray provides a robust and scalable platform capable of reliable discrimination between wild-type and mutant alleles, while simultaneously enabling multi-site detection within a single assay for mutation detection within clinically relevant *KRAS* hotspot regions in colorectal cancer.

**Table 3:** Synthetic oligonucleotide sequences targeting *KRAS* codons 12/13, 61, and 146, and their terminal modifications

Target gene	Oligo type		Sequence (5′–3′)
***KRAS*** ***Codon*** ***12/13***	Template	Wild-type	ATGACTGAATATAAACTTGTGGTAGTTGGAGCTGGTGGCGTAGGCAAGAGTGCCTTGACGATACAGCTAATTCAGAATCATTTTGTGGACGAATATGATC
		Codon 12 mutant (G12D)	ATGACTGAATATAAACTTGTGGTAGTTGGAGCTGATGGCGTAGGCAAGAGTGCCTTGACGATACAGCTAATTCAGAATCATTTTGTGGACGAATATGATC
	Primers and probes	Forward primer	ATGACTGAATATAAACTTGTGGTAGTTGGAGCT
		Reverse primer	Phosphorylated-ATCATATTCGTCCACAAAATGATTCTGAATTAG
		Wild-type inhibitor	GCCACC-**BHQ2**
		Fluorescent probe	**Cy5**-TCCAACTACCACAAGTT
		Capture probe	Biotin-GATCATATTCGTCCACAAAATGATTCTGAATTAG
***KRAS Codon 61***	Template	Wild-type	GTAGTAATTGATGGAGAAACCTGTCTCTTGGATATTCTCGACACAGCAGGTCAAGAGGAGTACAGTGCAATGAGGGACCAGTACATGAGGACTGGGGAGG
		Codon 61 mutation (Q61H)	GTAGTAATTGATGGAGAAACCTGTCTCTTGGATATTCTCGACACAGCAGGTCATGAGGAGTACAGTGCAATGAGGGACCAGTACATGAGGACTGGGGAGG
	Primers and probes	Forward primer	GTAGTAATTGATGGAGAAACCTGTCTCTTGGAT
		Reverse primer	Phosphorylated- CCTCCCCAGTCCTCATGTACTGGTCCCTCATTG
		Wild-type inhibitor	TTGACC-**BHQ2**
		Fluorescent probe	**Cy5-**GTCGAGAATATCCAA
		Capture probe	Biotin-CCTCCCCAGTCCTCATGTACTGGTCCCTCATTG
***KRAS Codon 146***	Template	Wild-type	CAGGCTCAGGACTTAGCAAGAAGTTATGGAATTCCTTTTATTGAAACATCAGCAAAGACAAGACAGGGTGTTGATGATGCCTTCTATACATTAGTTCGAG
		Codon 146 mutant (A146T)	CAGGCTCAGGACTTAGCAAGAAGTTATGGAATTCCTTTTATTGAAACATCAACAAAGACAAGACAGGGTGTTGATGAT GCCTTCTATACATTAGTTCGAG
	Primers and probes	Forward primer	CAGGCTCAGGACTTAGCAAGAAGTTAT
		Reverse primer	Phosphorylated- CTCGAACTAATGTATAGAAGGCATCATC
		Wild-type inhibitor	TGCTGA -**BHQ2**
		Fluorescent probe	**Cy5-** AATAAAAGGAATTCC
		Capture probe	Biotin-TAAGTCCTGAGCCTGTTTTGTGTCTACTGT

Wild-type and mutant bases are shown in blue and red, respectively. Terminal modifications include Cy5 (cyanine5), BHQ2 (Black Hole Quencher 2), biotin, and phosphate groups.

To further validate the high specificity of the 3D plasmonic *KRAS* microarray, we performed mutation detection in six colorectal cancer (CRC) cell lines with distinct *KRAS* codon mutations and one *KRAS* wild-type cell line. According to the literature, SW620 (G12V, c.35 G > T), SW1116 (G12A, c.35 G > C), and SNU-407 (G12D, c.35 G > A) each harbor codon 12 mutations; LoVo carries a codon 13 mutation (G13D, c.38 G > A); NCI-H460 harbors a codon 61 mutation (Q61H, c.183 A > T); and LS1034 possesses a codon 146 mutation (A146T, c.436 G > A). HT-29 is *KRAS* wild-type (Fig. 4b) gDNA from each cell line was amplified by multiplex blocked RPA and subsequently hybridized on the 3D plasmonic microarray. The microarray was designed so that each target codon (12/13, 61, and 146) is represented by a single probe spot, where one primer–probe set enables detection of all possible nucleotide substitutions within that codon region. To confirm this capability, we examined three codon 12 mutant cell lines (SW620, SW1116, SNU-407) and one codon 13 mutant cell line (LoVo). All four cell lines generated strong fluorescence exclusively at the codon 12/13 probe spot, with no cross-signal detected at other positions. Similarly, NCI-H460 and LS1034 showed distinct fluorescence only at the codon 61 and codon 146 probe spots, respectively, while HT-29 (wild-type) displayed no detectable signal.

These results clearly demonstrate that the 3D plasmonic microarray can distinguish *KRAS* mutant from wild-type sequences with high specificity. In particular, the ability to interrogate each clinically relevant codon (12, 13, 61, and 146) within a single probe spot using one primer–probe set simplifies assay design, reduces probe redundancy, and ensures comprehensive detection of mutational heterogeneity. This highlights the platform’s utility as a robust and multiplexed molecular diagnostic tool for ultrasensitive detection of *KRAS* mutations within clinically relevant hotspot regions in colorectal cancer.

### Clinical testing of tissue gDNA, plasma cfDNA, and urine cfDNA with the Plasmonic *KRAS* Microarray

**Fig. 5:**
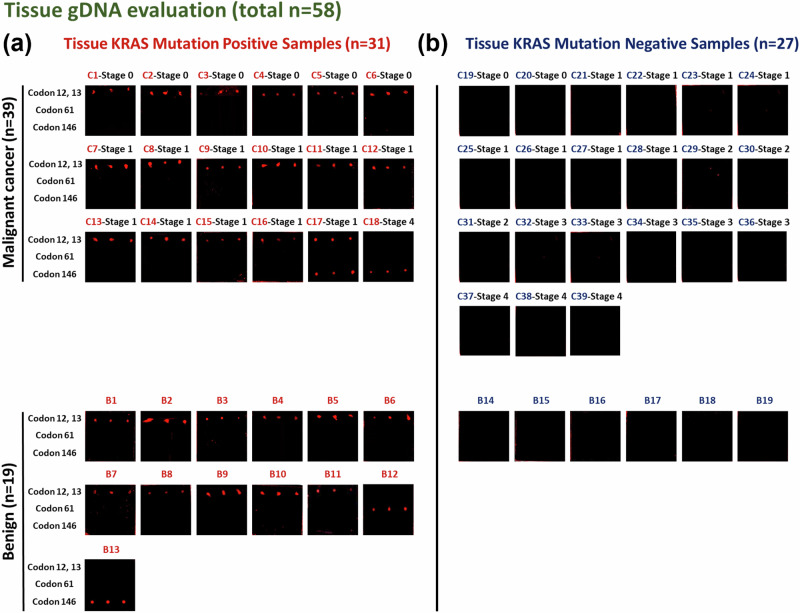
*KRAS* mutation profiling in tissue-derived genomic DNA (gDNA) from colorectal tumor samples (*n* = 58), including both malignant and benign tumors, using 3D plasmonic microarray targeting codons 12/13, 61, and 146. Results are compared with those obtained from a commercial tissue gDNA *KRAS* mutation detection kit. **a** Evaluation results of tissue gDNA samples identified as *KRAS* mutation-positive (*n* = 31) by the commercial kit. **b** Evaluation results of tissue gDNA samples identified as *KRAS* mutation-negative (*n* = 27) by the commercial kit.

Orthogonal validation across different clinical sample types is essential to establish the reliability of a mutation detection platform. In particular, cross-comparison between tissue-derived gDNA and minimally invasive liquid biopsy samples, such as plasma and urine cfDNA, provides critical evidence for clinical translation. To clinically validate the performance of the 3D plasmonic *KRAS* microarray, we analyzed paired tissue gDNA, plasma cfDNA, and urine cfDNA samples from colorectal tumor patients (*n* = 58). According to the commercial PNAClamp *KRAS* kit results, *KRAS* mutations were confirmed in 31 tissue cases and wild-type in 27 cases. When these gDNA samples were applied to the plasmonic microarray, all mutation-positive cases produced distinct codon-specific signals, while no signal was detected in mutation-negative cases, corresponding to 100% concordance with the tissue *KRAS* mutation status (Fig. 5, Table 4, and Table S3). Importantly, this perfect concordance was achieved regardless of tumor type, with both malignant and benign *KRAS* mutation-positive tissues yielding clear mutation signals, and all mutation-negative tissues showing no detectable fluorescence. These results confirm that the plasmonic microarray reliably captures codon-specific mutations directly from tissue-derived gDNA.

**Table 4:** Concordance rates of 3D-nanoplasmonic *KRAS* mutation microarray in tissue, plasma and urine samples with tissue *KRAS* mutation status in colorectal tumor patients

Tumor types	Tissue *KRAS* mutation Status		Concordance of 3D-nanoplasmonic *KRAS* microarray (%)		
			Tissue	Plasma	Urine
**Malignant colorectal tumor (*n*** = **39)**	Mutation positive	*n* = 18	**100% (18/18)**	**100% (18/18)**	**100% (18/18)**
	Mutation negative	*n* = 21	**100% (21/21)**	**85.7% (18/21)**	**100%(21/21)**
**Benign colorectal tumor (*n*** = **20)**	Mutation positive	*n* = 13	**100% (13/13)**	**15% (2/13)**	**8% (1/13)**
	Mutation negative	*n* = 6	**100% (6/6)**	**100% (6/6)**	**100% (6/6)**

**Fig. 6:**
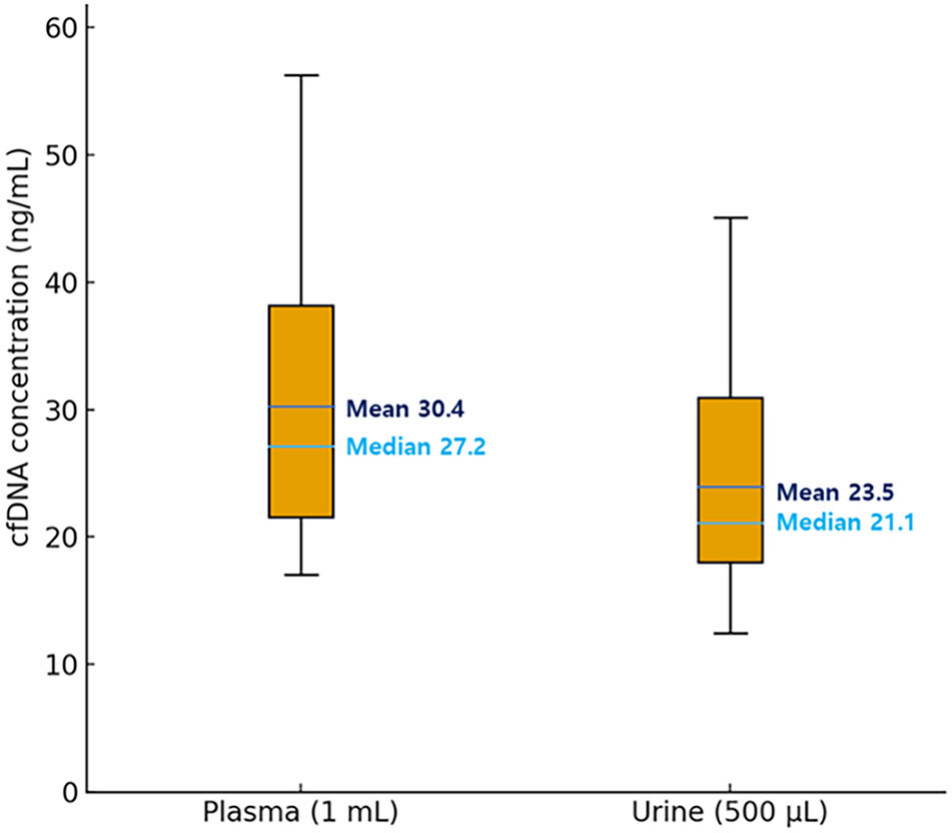
Boxplot comparison of cfDNA yields from plasma and urine. Plasma samples (1 mL) and urine samples (500 μL) were analyzed for cfDNA concentration.

For plasma cfDNA analysis, 500 µL of plasma was extracted and eluted in 30 µL, yielding an average cfDNA concentration of 30.4 ng/µL, and 1 µL of the eluate was used per analysis (Fig. 6). Agarose gel analysis of DNA extracted from plasma of colorectal cancer patients revealed a distinct band in the 100–200 bp range, consistent with the characteristic cfDNA fragment size (~160–180 bp). Additional bands were observed in the 400–500 bp region and above 2000 bp, suggesting the presence of di-nucleosomal fragments and a certain amount of high-molecular-weight genomic DNA (Fig. S9). These higher-molecular-weight fragments are likely attributable to partial leukocyte lysis during sample handling and storage. Accordingly, the samples are interpreted as containing a mixture of cfDNA fragments and high-molecular-weight DNA.

**Fig. 7:**
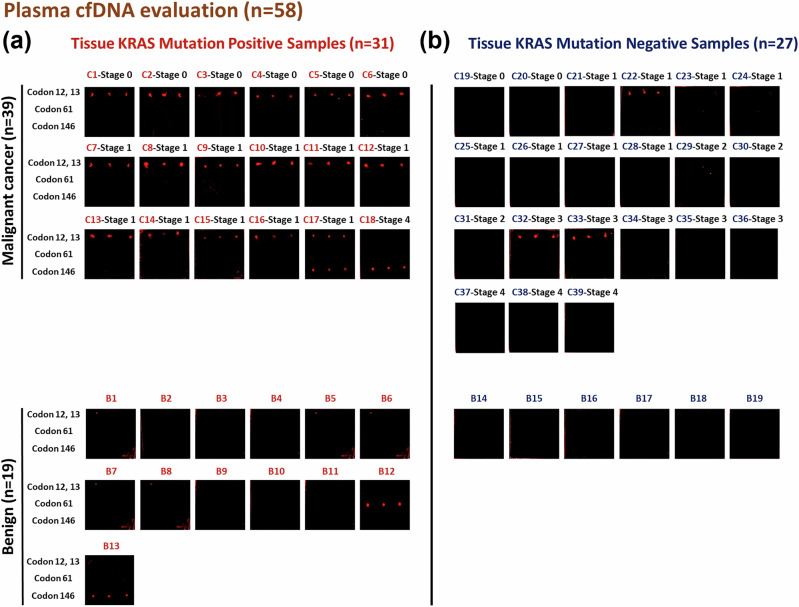
*KRAS* mutation profiling in plasma cfDNA matched to tissue gDNA from colorectal tumor samples (*n* = 59), including both malignant and benign tumors, using 3D plasmonic microarray. **a** Evaluation results of plasma cfDNA samples matched to tissue *KRAS* mutation-positive cases (*n* = 31). **b** Evaluation results of plasma cfDNA samples matched to tissue *KRAS* mutation-negative cases (*n* = 27).

All 18 malignant tissue-positive cases were consistently detected (100%), while 18 of 21 malignant tissue-negative cases were correctly classified, resulting in an overall concordance of 85.7% (Fig. 7, Table 4, and Table S3). Three malignant tissue-negative cases showed positive signals in plasma cfDNA, which may represent false positives, but could also reflect tumor heterogeneity or spatial variation between tissue biopsy and circulating DNA. Among benign cases, only 2 of 13 mutation-positive samples were detected in plasma (15.4%), whereas all six benign mutation-negative samples were correctly classified (100%). The reduced detection rate in benign tumors likely reflects their slower proliferation and lower levels of ctDNA release into circulation, which may act conservatively by minimizing the risk of overestimation.

**Fig. 8:**
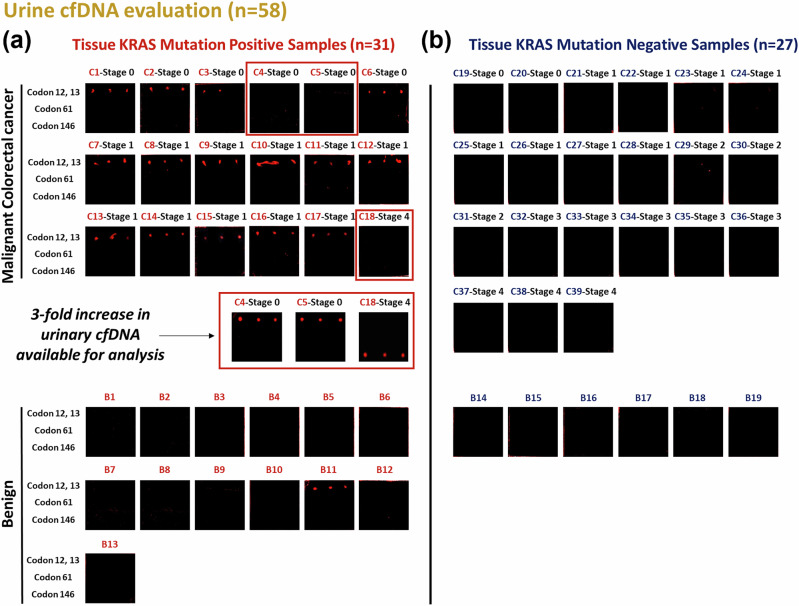
*KRAS* mutation profiling in urinary cfDNA matched to tissue gDNA from colorectal tumor samples (*n* = 59), including both malignant and benign tumors, using 3D plasmonic microarray. **A** Evaluation results of urinary cfDNA samples matched to tissue *KRAS* mutation-positive cases (*n* = 31). **B** Evaluation results of urinary cfDNA samples matched to tissue *KRAS* mutation-negative cases (*n* = 27).

For urine cfDNA, 1 mL of urine was extracted and eluted, yielding an average concentration of 23.7 ng/µL, and 1 µL of the eluate was used per analysis (Fig. 6). Using this input, 15 of 18 malignant mutation-positive cases were detected (83.3%). When the urine cfDNA input was increased three-fold, all malignant mutation-positive (18/18) and mutation-negative (21/21) cases were correctly classified, yielding 100% concordance with tissue status (Fig. 8, Table 4, and Table S3). In benign cases, all mutation-negative samples were accurately classified (100%), while only 1 of 13 mutation-positive samples (7.7%) was detected.

Although Stage 0 (carcinoma in situ) lesions are histologically confined to the epithelial layer and typically lack overt vascular invasion, the release of tumor-derived DNA into the circulation does not necessarily require macroscopically evident vascular infiltration. Circulating DNA fragments in cancer patients are known to arise predominantly from apoptotic and necrotic tumor cells, suggesting that fragmented DNA may be released into surrounding stromal compartments even in the absence of invasive growth. In addition, circulating tumor DNA has been reported in patients with early-stage cancers, indicating that low-level tumor DNA shedding can occur prior to clinically apparent invasion^33,34^. Accordingly, the low-frequency mutation signals observed in Stage 0 patients in this study may reflect minimal but biologically plausible tumor DNA release. The precise mechanism underlying this observation remains to be clarified.

In mutation-positive benign CRC cases, relatively low concordance was observed between tissue and biofluid (plasma and urine) detection outcomes. Tissue samples were collected during colonoscopic biopsy, and matched plasma and urine samples were obtained on the same day, minimizing the likelihood that temporal variation contributed to the discrepancy. The reduced concordance is therefore more plausibly attributed to biological factors. Benign CRC lesions generally exhibit smaller tumor burden and lower cellular turnover compared to malignant tumors, which may substantially limit the release of tumor-derived cfDNA into circulation. As a result, the variant allele frequency (VAF) in plasma and urine is expected to be extremely low. Under such ultra-low VAF conditions, mutant alleles may fall below the practical detection threshold in biofluids, even when using highly sensitive analytical platforms. Accordingly, the observed discordance between tissue and biofluid results likely reflects limited cfDNA shedding from benign lesions rather than analytical performance limitations of the 3D plasmonic microarray assay.

Together, these clinical results demonstrate that the plasmonic *KRAS* microarray enables accurate detection of clinically relevant *KRAS* hotspot mutations across tissue, plasma, and urine samples. In malignant colorectal cancer patients, the platform achieved 100% concordance between tissue and plasma as well as tissue and urine cfDNA when sufficient cfDNA input was applied. While the sample size was limited, these results suggest robust performance within the evaluated cohort. By contrast, in benign colorectal tumors, plasma and urine cfDNA showed very low detection rates despite tissue-confirmed mutations, likely reflecting minimal ctDNA shedding. This sharp distinction highlights the high specificity of the platform and its potential utility in differentiating clinically significant mutations. Furthermore, the results emphasize that cfDNA input optimization is critical—as demonstrated in urine cfDNA, where increased input restored complete concordance with tissue status. Collectively, these findings establish the plasmonic *KRAS* microarray as a highly sensitive and specific platform with strong translational potential for non-invasive *KRAS* mutation testing across multiple clinical sample types.

## Discussion

Accurate detection of *KRAS* mutations remains a major clinical need in colorectal cancer, given their importance in guiding anti-*EGFR* therapy and monitoring disease progression. Yet, detecting mutations in cfDNA continues to be technically challenging due to their extremely low abundance and the overwhelming background of wild-type sequences. Commercial assays illustrate these limitations: PNAClamp™ failed to detect plasma cfDNA mutations despite tissue positivity, and although ADPS™ improved sensitivity, its high false-positive rate raised concerns about specificity. These shortcomings emphasize the necessity of novel diagnostic approaches that combine high sensitivity and specificity with clinical practicality.

The 3D plasmonic *KRAS* microarray presented here integrates blocked RPA + hybridization on a plasmonic microarray to overcome these challenges. This approach suppresses wild-type background while amplifying mutant-specific signals, enabling detection of codon-level *KRAS* mutations at variant frequencies as low as 10⁻⁹—substantially surpassing the 0.1–1% limits of conventional PCR and the ~0.01% range achievable with advanced NGS. The ability to interrogate entire codons with a single primer–probe set further simplifies assay design while maintaining high analytical specificity, as demonstrated using both synthetic templates and colorectal cancer cell lines.

Table 5 summarizes representative plasmonic-based mutation detection platforms and wild-type suppression strategies. While surface plasmon resonance imaging (SPRI)^31^ and localized surface plasmon resonance (LSPR) absorbance peak shift systems^32^ enable label-free or simplified detection, their analytical sensitivity and quantitative characterization remain relatively constrained. Gold nanoparticle- and CRISPR-based metal-enhanced fluorescence (MEF)^33^, as well as PCR-assisted surface-enhanced Raman scattering (SERS)^34^, have improved detection limits. Furthermore, several platforms incorporating wild-type suppression strategies have been reported. The electrochemical PNA clamp PCR platform^35^ employs hybridization-mediated wild-type blocking coupled with electrochemical detection, achieving a reported sensitivity of 1 fg/μL (0.01% mutant fraction). Similarly, the LNA-based SERS platform^33^ enriches mutant targets via locker probe–mediated wild-type sequestration, followed by magnetic bead purification and SERS detection, achieving a sensitivity of 100 copies per reaction. Nevertheless, many of these platforms still encounter challenges in achieving robust low-VAF discrimination and scalable multiplex implementation.

**Table 5:** Comparison of representative plasmonic mutation detection platforms and wild-type suppression strategies

Ref #	Detection method	Amplification strategy	Limit of detection	Variant allele frequency (VAF) sensitivity	Assay time
^33^	Surface plasmon resonance imaging (SPRI)	PCR-free hybridization	~300 copies	Not reported	2.5 h
^36^	Localized surface plasmon resonance (LSPR) absorbance peak shift	PCR-free hybridization	2 ng/mL	Not reported	1 h
^37^	Au nanoparticle-assisted metal-enhanced fluorescence	CRISPR/Cas12a	1 fM	Not reported	30 min
^38^	Surface-enhanced Raman scattering (SERS)	PCR	10 copies	0.1%	1.5 h
^39^	Electrochemical detection	PNA Clamp PCR	1 fg/μL	0.01%	1.5 h
^40^	Surface-enhanced Raman scattering (SERS)	Locked nucleic acid (LNA) PCR + magnetic bead-based purification	100 copies	Not reported	30 min
**This study**	**Plasmon-enhanced fluorescence (PEF)**	**Wild-type blocked RPA** **+** **wild-type signal suppression**	**100** **zM (3 copies)**	**10**^**−9**^**%**	1 h

In contrast, the present study introduces a dual-stage wild-type suppression architecture consisting of (i) selective inhibition of wild-type amplification during the RPA enzymatic step and (ii) secondary signal suppression using a quencher during the hybridization/detection stage. By structurally reducing wild-type amplification prior to signal generation and further suppressing background signals during detection, our strategy enhances the mutant signal-to-background ratio. Moreover, integration with plasmon-enhanced fluorescence (PEF) readout enables continuous allele discrimination across both amplification and detection stages, thereby mechanistically differentiating our system from previously reported hybridization-based suppression platforms. In contrast, this study employs a wild-type–specific quencher probe to suppress wild-type amplification and minimize background fluorescence, while mutant codons are preferentially amplified and enhanced on the plasmonic substrate. By targeting the wild-type sequence rather than individual mutant variants, the platform enables detection of all possible mutations within a codon region. Notably, the proposed system achieves the highest analytical sensitivity among the compared plasmonic platforms, supporting its applicability for circulating tumor DNA analysis, particularly in low-frequency mutation detection.

Clinical validation provided strong evidence for translational potential. In malignant colorectal cancer patients, *KRAS* mutation-positive tissues identified by the MFDS-approved PNAClamp™ kit showed 100% concordance with results obtained from the plasmonic *KRAS* microarray across tissue, plasma, and urine cfDNA when sufficient cfDNA input was used, confirming that the assay can reliably translate tissue mutation profiles into liquid biopsy readouts. In benign tumors, however, plasma and urine cfDNA detection rates were markedly lower (15.4 and 7.7%, respectively) despite tissue-confirmed mutations. This discrepancy likely reflects biological differences such as slower cell turnover and minimal ctDNA release in benign lesions, which may act conservatively by reducing the risk of overestimation. Importantly, cfDNA input volume was also shown to be critical; increasing urine cfDNA input restored complete concordance with tissue results, highlighting the importance of assay optimization for reliable detection in low-abundance settings such as early-stage cancers.

Collectively, these findings demonstrate that the plasmonic *KRAS* microarray provides ultra-sensitive, highly specific, and versatile mutation detection across tissue, plasma, and urine specimens. Its ability to achieve perfect concordance in malignant cases, alongside its conservative behavior in benign tumors, highlights both its diagnostic accuracy and its clinical safety margin. By addressing the technical limitations of PCR- and NGS-based platforms, this technology shows strong promise as a non-invasive, cost-effective tool for early colorectal cancer detection, therapeutic decision-making, and minimal residual disease monitoring.

## Methods

### Clinical sample and clinical information collection

Colorectal cancer patients were prospectively enrolled at Samsung Medical Center (Seoul, Korea) between 2021 and 2024 under Institutional Review Board approval (IRB No. SMC 2021-06-083-015). Written informed consent was obtained from all participants prior to sample and data collection. All procedures involving human participants were conducted in accordance with the Declaration of Helsinki. Anonymized clinical samples were transferred to the Korea Institute of Materials Science (KIMS), where all analyses were conducted in compliance with relevant ethical guidelines. Collected clinical information included age, sex, and diagnostic findings. Tissue, blood, and urine samples were obtained from each participant. Tissue samples for research were collected only when sufficient material was available following clinical diagnosis or surgical resection without prior biopsy. No samples were taken when the lesion was too small or entirely required for clinical purposes, and no additional procedures were performed solely for research. For research purposes, up to 20 mL of whole blood and ~50 mL of urine were collected. Tissue samples were collected in sterile tubes and stored at below –80 °C until analysis. Whole blood (up to 20 mL) was drawn into EDTA-treated vacuum tubes, stored at 4 °C, and centrifuged within 8 h (1600×*g*, 10 min, 4 °C). Plasma was separated, transferred to sterile tubes, and stored at below –80 °C until analysis. Urine was collected by the patient into a paper cup, transferred to dedicated tubes, aliquoted into 10 mL portions within 5 h, and stored at below −80 °C until analysis. Clinical classification of colorectal cancer was based on histopathological biopsy results. Patients were categorized as benign or malignant (stage 0, I, II, III, or IV) according to the clinical diagnosis.

### Evaluation of *KRAS* mutation detection with a commercial kit for comparative analysis

*KRAS* mutations in colorectal cancer tissues (benign and malignant) were analyzed by HLB Panagene Inc. (Korea), a commercial genetic analysis company. Genomic DNA (gDNA) was extracted using the PANAMAX™ Tissue Cultured Cells DNA Extraction Kit (HLB Panagene Inc., Korea) using the PANAMAX 48 extraction instrument. *KRAS* mutations at codons 12, 13, 59, 61, 117, and 146 were analyzed using the PNAClamp™ *KRAS* Mutation Detection Kit (Ver.4) (HLB Panagene Inc.), which is Conformité Européenne - In Vitro Diagnostic (CE-IVD) certified and approved by the Korean Ministry of Food and Drug Safety (MFDS) for use with tissue biopsy samples, on a Bio-Rad CFX96™ Real-time PCR system. Samples determined to be *KRAS* mutation-positive were further analyzed by Sanger sequencing (SolGent Inc., Korea) to identify the specific mutation types.

Cell-free DNAs (cfDNAs) extracted from plasma samples were also analyzed using the same method with the PNAClamp™ Mutation Detection Kit ver.4. In addition, plasma cfDNAs were further analyzed using the ADPS™ *KRAS* Mutation Test Kit (HLB Panagene Inc.) as a research-use-only (RUO) product.

### Extraction of genomic DNA (gDNA) from colorectal cancer cell lines

Seven human colorectal cancer cell lines—HT-29, SW620, NCI-H460, LS1034, SNU-407, SW1116, and LOVO—were procured from the Korean Cell Line Bank (Seoul National University, Korea). The cells were cultivated in RPMI 1640 medium containing 10% fetal bovine serum (FBS) and penicillin under a humidified incubator environment set at 37 °C with 5% CO₂. Once the cultures approached 80% confluency, the cells were detached using a trypsin-EDTA solution and subsequently washed several times with Dulbecco’s phosphate-buffered saline (DPBS) to remove residual enzymes and media. Genomic DNA was isolated from the collected cells using the AccuPrep® Genomic DNA Extraction Kit (Bioneer, Daejeon, Korea), following the manufacturer’s instructions. DNA concentration and purity were assessed with a NanoPhotometer® (Implen, USA), and the samples were preserved at −80 °C until downstream use.

### Extraction of cell-free DNA (cfDNA) from plasma and urine

cfDNA was extracted from 500 µL of plasma and 1 mL of urine using the commercial MagListo™ cfDNA Extraction Kit (Bioneer, Daejeon, South Korea) following the manufacturer’s protocol, for subsequent analyses.

### Preparation of a 3D-nanoplasmonic substrate

A polyethylene terephthalate (PET) film with a thickness of 188 μm was procured from Toray Industries (Tokyo, Japan). A 97% solution of 1H,1H,2H,2H-perfluorodecanethiol (PFDT) was purchased from Sigma-Aldrich (St. Louis, MO, USA). Gold (Au) was obtained from iTASCO (Seoul, Republic of Korea). The 3D-nanoplasmonic substrate was prepared according to a previously reported method by our group^21^. In brief, the PET film underwent argon plasma treatment for 2 min using a custom-built radio frequency (RF) ion-etching system (LAT CO.,LTD., Gyeonggi-do, Republic of Korea) under fixed conditions: 5 sccm Ar gas flow, 80 mTorr chamber pressure, and plasma power set to 100 W. A gold (Au) layer was deposited onto the PET nanopillars at a deposition rate of 2.0 Å per second, maintained under a base pressure of 9.6 × 10^−^⁶ Torr, using a thermal evaporation apparatus. Following this, the substrate was treated with PFDT, after which an additional Au layer was thermally evaporated onto the PFDT-coated Au/PET nanopillar surface at a slower rate of 0.3 Å/s.

### Oligonucleotide design and synthesis

Synthetic oligonucleotides, including DNA templates, primers, and probes, were custom-synthesized by Bioneer (Daejeon, South Korea). The sequences and terminal modifications are listed in Table 1.

### DNA probe immobilization on 3D-nanoplasmonic substrates

Biotinylated capture probes (10 μM), specific to *KRAS* codons 12/13, 61, and 146, were individually mixed with streptavidin (0.1 mg/mL) and spotted onto a 3D-nanoplasmonic substrate using the BIOSPOT Custom system (BioFluidix, Germany) in a 3 × 3 array format. Each probe–streptavidin mixture was deposited in triplicate along a row, with 50nL droplets spaced at 3 mm intervals. The substrate was incubated at 4 °C to enable stable immobilization through gold–streptavidin–biotin interactions, followed by a water rinse to remove unbound materials. The remaining surface was blocked with 0.5% bovine serum albumin (BSA) for 2 h at 25 °C, washed to remove excess BSA, and stored at 4 °C until further use. Prior to hybridization, the 3D-nanoplasmonic microarray was affixed to a glass slide using double-sided adhesive tape to ensure stable positioning. A polydimethylsiloxane (PDMS) chamber was then attached over the array region to hold more than 50 μL of solution.

### *KRAS* mutation detection using RPA-coupled hybridization on 3D-nanoplasmonic microarray

Isothermal amplification was performed using the TwistAmp Liquid Basic kit (TwistDx, UK) in a 20 μL reaction mixture containing extracted gene, 500 nM of each primer and 10 μM of wild-type-specific blocker. The reaction was incubated at 39 °C for 30 min using a ThermoMixer C (Eppendorf, Germany) under constant temperature conditions. To generate single-stranded DNA (ssDNA), 1 μL of the RPA product was treated with 1 μL of lambda exonuclease (Thermo Fisher Scientific, Carlsbad, CA, USA) and 5 μL of 10× reaction buffer. The total volume was adjusted to 50 μL with distilled water, and the mixture was incubated at 37 °C for 30 min. After digestion, 1 μL of a fluorescently labeled probe (5 μM) and 1 μL of wild-type inhibitor (500 μM) were added directly to the 50 μL reaction mixture. The final solution was applied to the 3D-nanoplasmonic microarray, sealed, and incubated at 37 °C for 30 min. Following incubation, the microarray was gently rinsed three times with distilled water to remove unbound materials and allowed to air dry completely prior to fluorescence analysis. For fluorescence image analysis, the PDMS chamber was removed from the 3D-nanoplasmonic microarray mounted on the glass slide prior to scanning. Fluorescence images were acquired using the InnoScan 710 microarray scanner (Innopsys, Carbonne, France) under identical conditions for all samples, including negative controls on the same slide. When no fluorescent signal was observed, brightness was increased to 100 to confirm the absence of background fluorescence. Final images were uniformly adjusted for contrast using the Innopsys analysis software to clearly display fluorescence signals.

## Supplementary information

Supplementary information

## Data Availability

The datasets generated and/or analyzed during the current study are not publicly available due to ethical restrictions associated with clinical data, but are available from the corresponding author on reasonable request.
