# Supplementary material for: Translational feasibility of a plasmonic microarray–based liquid biopsy for KRAS codon mutation detection across tissue, plasma, and urine in early colorectal cancer
Source: NPJ Precis Oncol. 2026 May 2;10:261. doi: 10.1038/s41698-026-01452-8 (PMC13342606; doi:10.1038/s41698-026-01452-8)
Supplement: Supplementary file 1 — Supplementary information [file 41698_2026_1452_MOESM1_ESM.docx]

Supplementary Information

Translational Feasibility of a Plasmonic Microarray–Based Liquid Biopsy for KRAS Codon Mutation Detection across Tissue, Plasma, and Urine in Early Colorectal Cancer

Ji Young Lee^1^, ChaeWon Mun,^1^ Eun Ran Kim,^2^ Sung-Gyu Park^1*^, Min-Young Lee^1*^

^1^Advanced Bio and Healthcare Materials Research Division, Korea Institute of Materials Science (KIMS); 797, Changwon-daero, Seongsan-gu, Changwon-si, Gyeongsangnam-do, 51508, Republic of Korea

^2^Division of Gastroenterology, Department of Medicine, Samsung Medical Center, Sungkyunkwan University School of Medicine, Seoul, Korea.

*Corresponding authors.

E-mail: [sgpark@kims.re.kr](mailto:sgpark@kims.re.kr) (Sung-Gyu Park), [myay0615@kims.re.kr](mailto:myay0615@kims.re.kr) (Min-Young Lee)

**Figure S1. Scanning electron microscopy (SEM) characterization of the 3D nanoplasmonic substrate.**

| **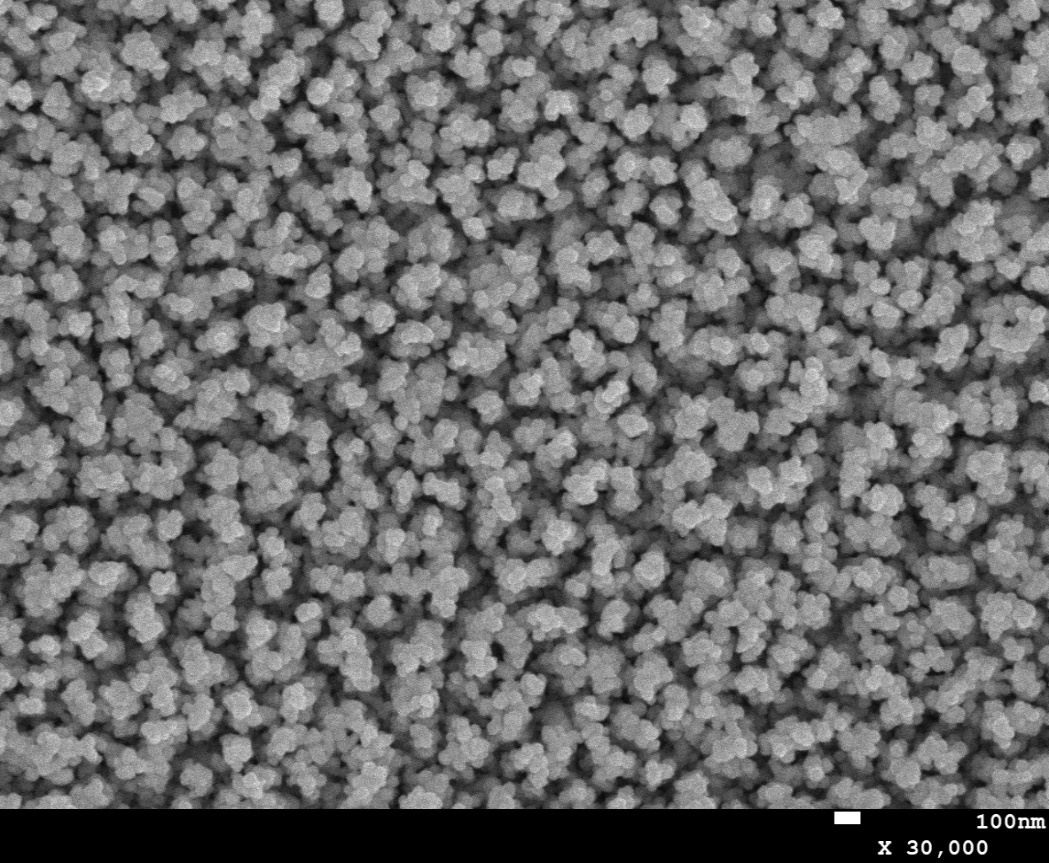** |
| --- |
| 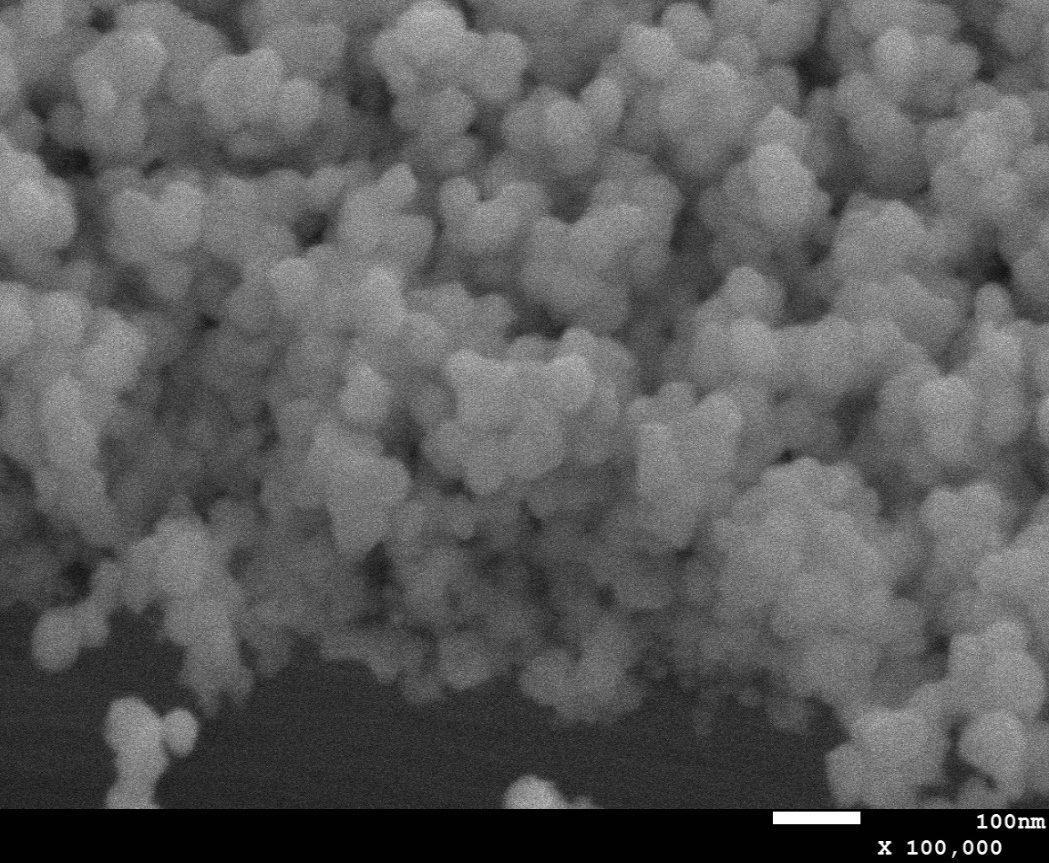 |

**Figure S2. Photograph of the 3D nanoplasmonic microarray chip for multiplex KRAS mutation detection.**


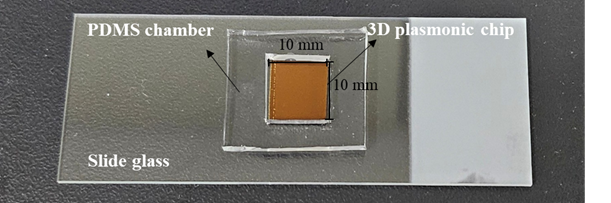


**Figure S3. Optimization of microarray hybridization time and wild-type blocker concentration.** (a) Fluorescence images obtained after surface hybridization at different incubation times (10, 20, 30, and 40 min) using 100 nM template. (b) Evaluation of wild-type blocker quencher probe concentration (5, 10, and 15 μM) under 100 nM wild-type template conditions. (c) Fluorescence detection of mutant template down to 1 fM in the presence of 10 μM wild-type blocker.

**(a)**


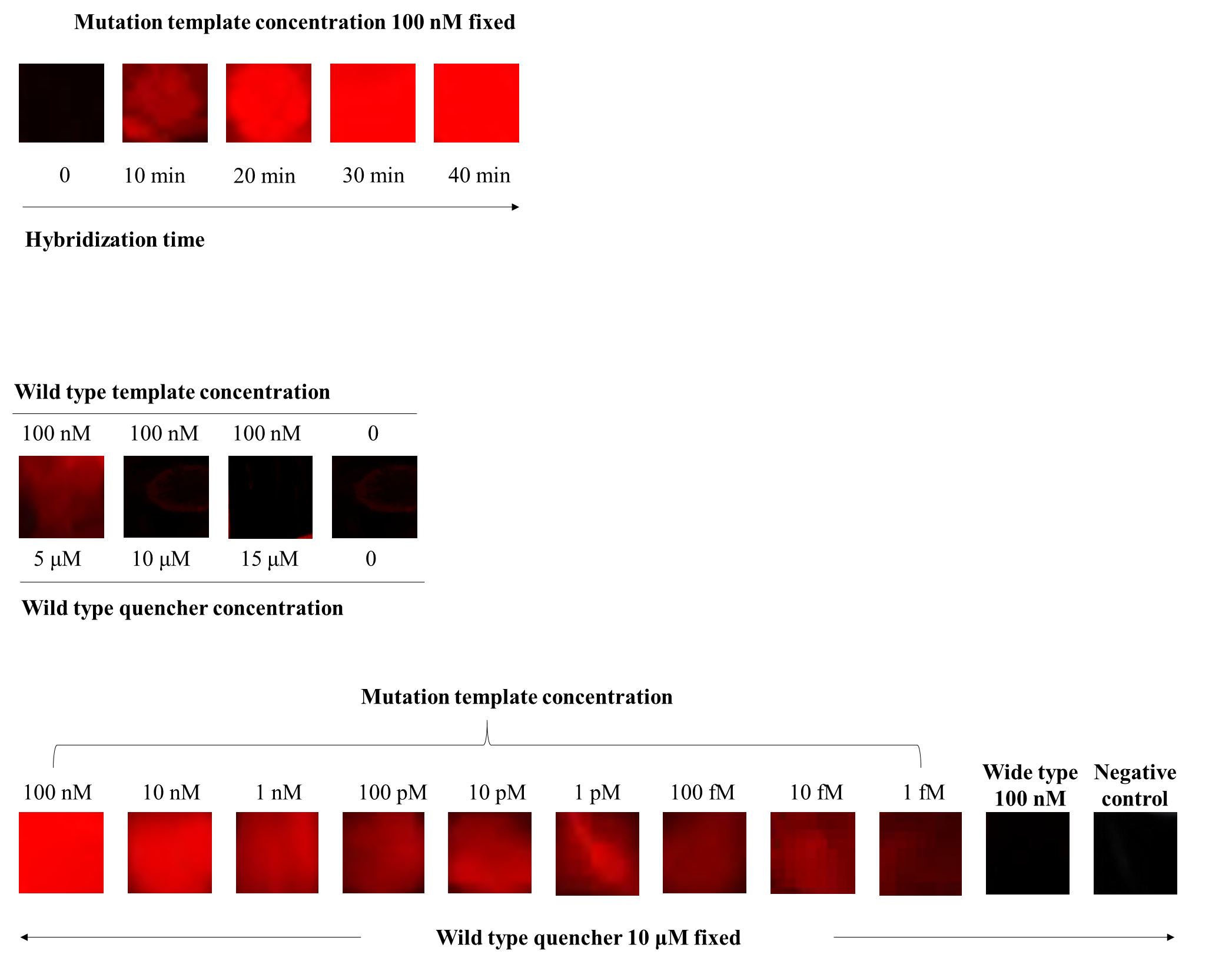


**(b)**

**(d)**

**Figure S4. Linearity assessment of the nanoplasmonic fluorescence assay.** Fluorescence intensity was measured after hybridization on the nanoplasmonic substrate using nine concentration points of KRAS mutant DNA template spanning from 100 aM to 1 pM.

**
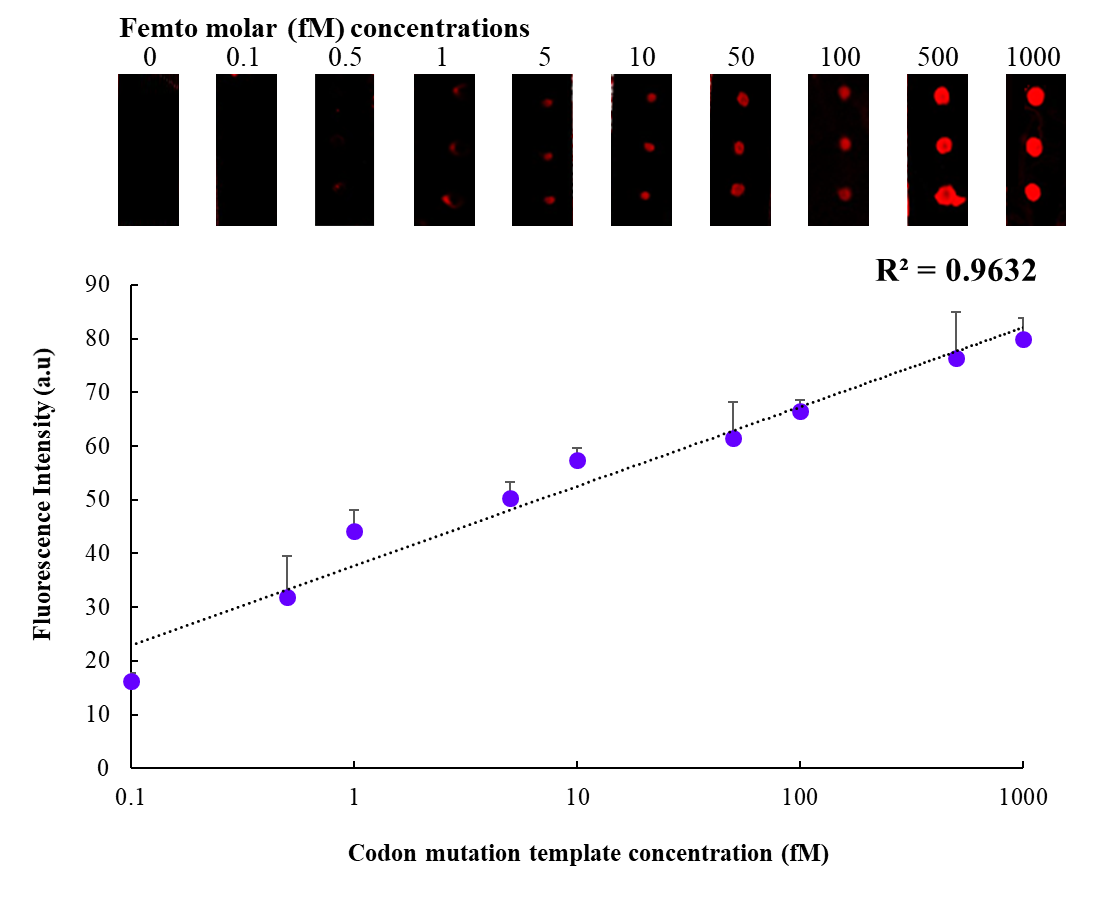
**

**Figure S5. Evaluation of wild-type amplification suppression by the quencher-labeled inhibitor.** (a) Real-time PCR amplification curves of mutant and wild-type templates in the presence of 10 μM wild-type inhibitor. (b) Ct values plotted as a function of log template concentration. (c) Comparative Ct values of mutant and wild-type templates under inhibition conditions.

**
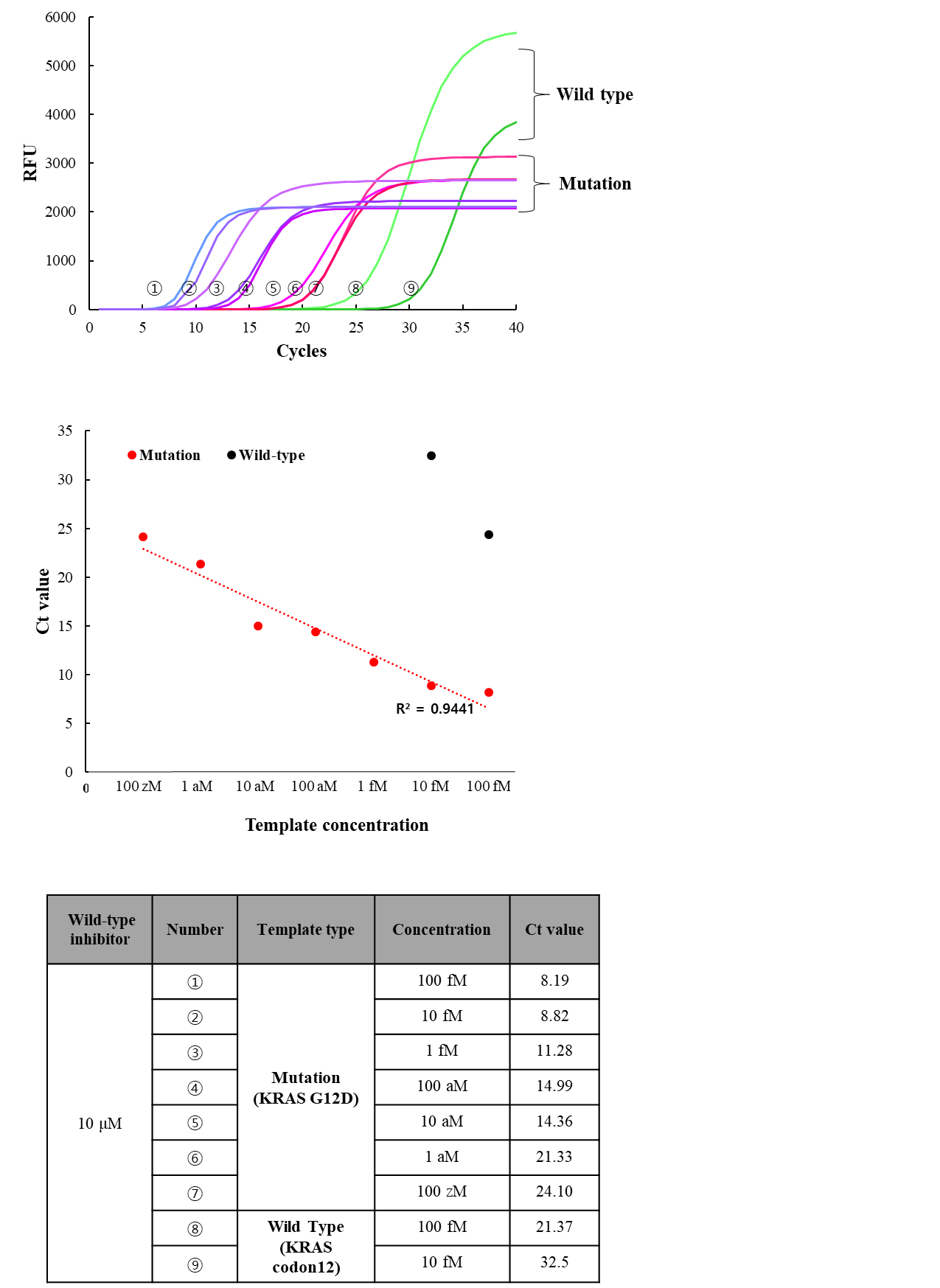
**

**(b)**

**(c)**

**(a)**

**Figure S6. Reproducibility of the complete blocked RPA–plasmonic microarray assay at 500 zM (15 copies) under excess wild-type background (10 nM WT DNA).**


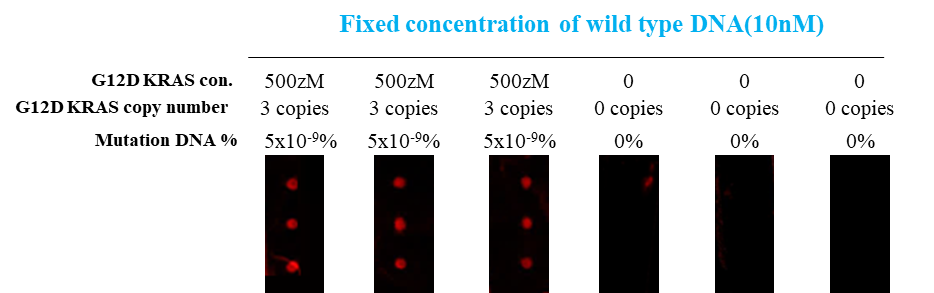


**Figure S7. Detection performance after cfDNA extraction in biological matrices.** Assay performance in (a) fetal bovine serum (FBS) and (b) healthy human urine spiked with 100-mer mutant ssDNA templates (100 fM–100 zM) following cfDNA extraction.

**
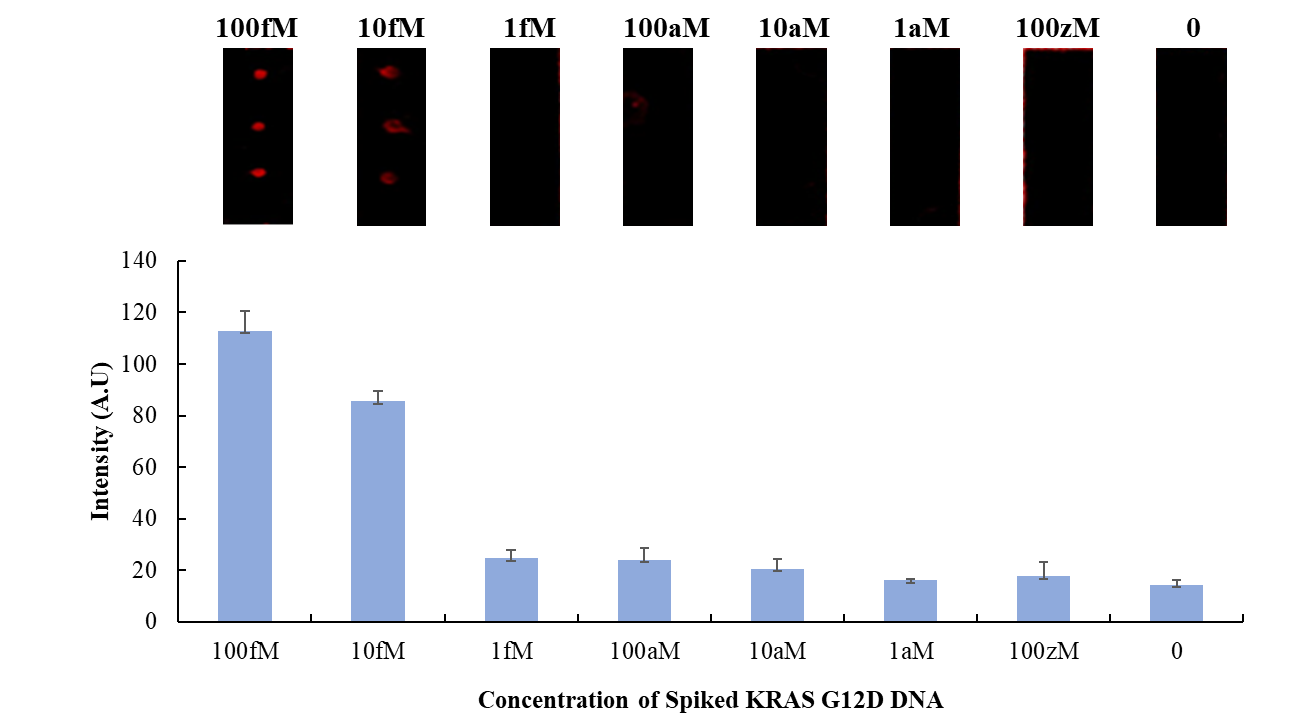
**

**(a)**

**(b)**

**
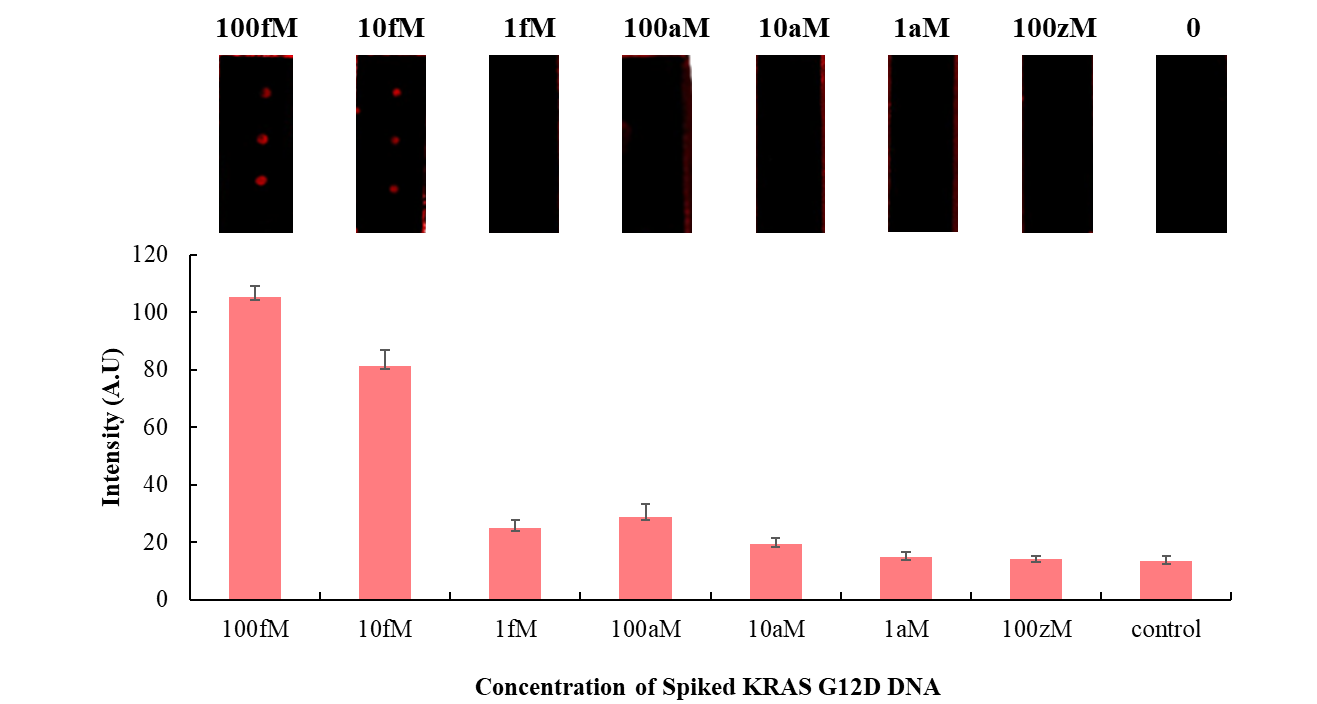
**

**Figure S8. Storage stability of streptavidin–biotin-functionalized plasmonic microarrays after one week of dry storage at 4°C.** Representative fluorescence images and quantitative fluorescence intensity (a.u.) were obtained after hybridization.

**
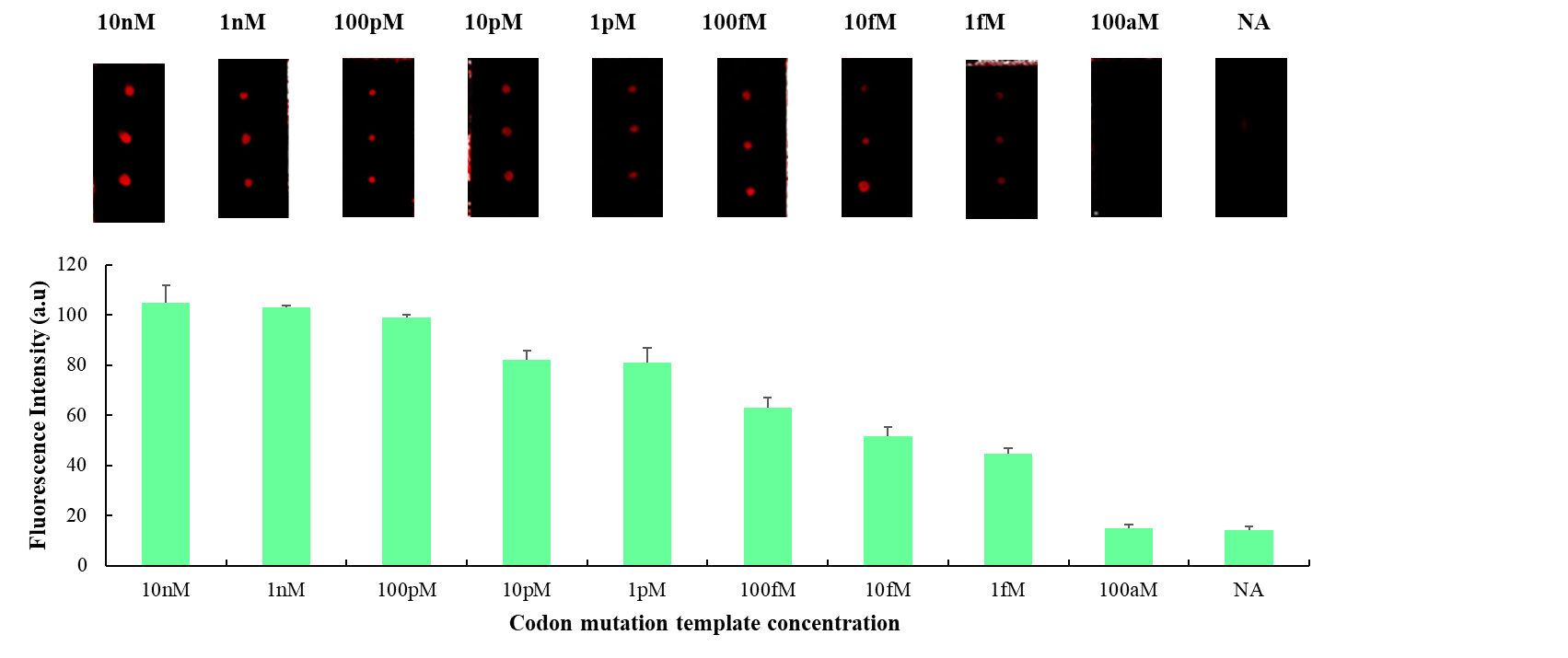
**

**Figure S9. cfDNA fragment size analysis confirming minimal genomic DNA contamination.**  Agarose gel electrophoresis of cfDNA extracted from colorectal cancer samples showed the expected cfDNA fragment size distribution (~150–200 bp), while high molecular-weight genomic DNA (gDNA) bands/smears were also observed, indicating gDNA contamination.


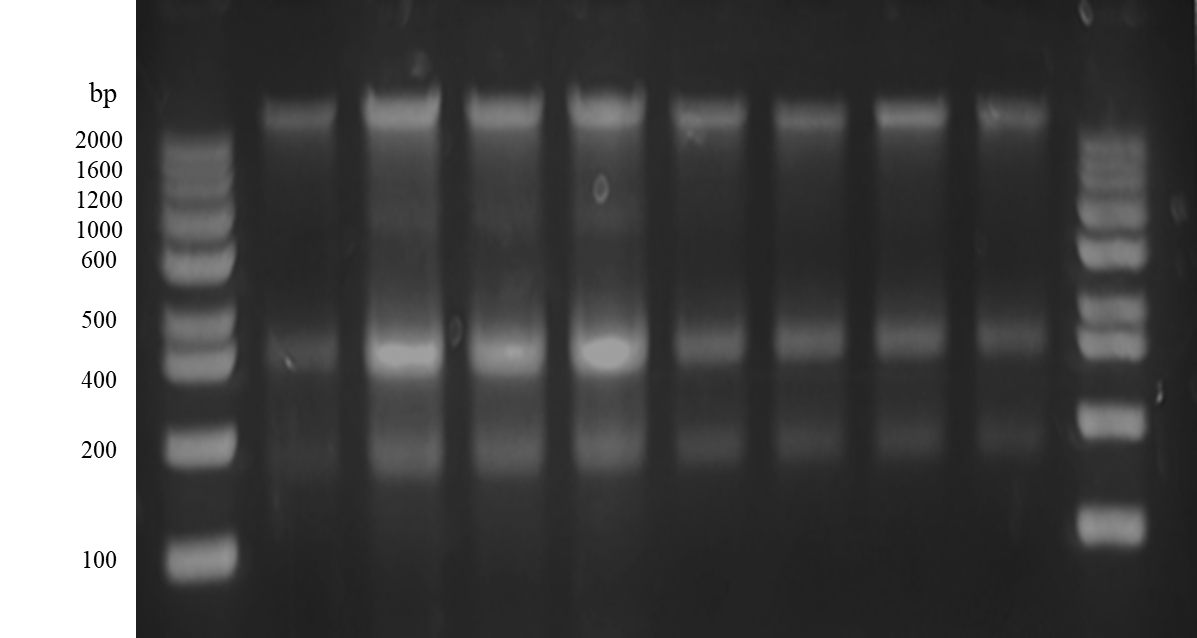


**Table S1. Clinical characteristics of the patients with colorectal tumors involved in this study.**

| **Classification** | **Patient number** | **Age** | **Sex** | **Histological type** | **Pathologic**  **stage** | **CEA** |
| --- | --- | --- | --- | --- | --- | --- |
| **Malignant colorectal tumor** | C1 | 64 | F | Adenocarcinoma | Stage 0 | 1.98 |
|  | C2 | 54 | F | Adenocarcinoma | Stage 0 | - |
|  | C3 | 64 | M | Adenocarcinoma | Stage 0 | - |
|  | C4 | 77 | F | Adenocarcinoma | Stage 0 | - |
|  | C5 | 44 | F | Adenocarcinoma | Stage 0 | - |
|  | C6 | 67 | F | Adenocarcinoma | Stage 0 | - |
|  | C7 | 56 | F | Adenocarcinoma | Stage Ⅰ | - |
|  | C8 | 66 | F | Adenocarcinoma | Stage Ⅰ | 4.07 |
|  | C9 | 70 | F | Adenocarcinoma | Stage Ⅰ | 1.53 |
|  | C10 | 57 | M | Adenocarcinoma | Stage Ⅰ | - |
|  | C11 | 25 | M | Adenocarcinoma | Stage Ⅰ | - |
|  | C12 | 67 | F | Neuroendocrine tumor | Stage Ⅰ | 1.51 |
|  | C13 | 67 | M | Adenocarcinoma | Stage Ⅰ | 1.15 |
|  | C14 | 61 | M | Adenocarcinoma | Stage Ⅰ | 3.13 |
|  | C15 | 63 | F | Adenocarcinoma | Stage Ⅰ | 0.49 |
|  | C16 | 67 | F | Adenocarcinoma | Stage Ⅰ | 1.78 |
|  | C17 | 71 | M | Adenocarcinoma | Stage Ⅰ | 3.76 |
|  | C18 | 57 | M | Adenocarcinoma | Stage ⅠV | 0.68 |
|  | C19 | 70 | F | Adenocarcinoma | Stage 0 | - |
|  | C20 | 70 | F | Adenocarcinoma | Stage 0 | 5.84 |
|  | C21 | 58 | M | Adenocarcinoma | Stage Ⅰ | 2.68 |
|  | C22 | 50 | F | Adenocarcinoma | Stage Ⅰ | 2.35 |
|  | C23 | 78 | M | Adenocarcinoma | Stage Ⅰ | 3.69 |
|  | C24 | 67 | M | Adenocarcinoma | Stage Ⅰ | 2.72 |
|  | C25 | 68 | M | Adenocarcinoma | Stage Ⅰ | 2.66 |
|  | C26 | 58 | F | Adenocarcinoma | Stage Ⅰ | 0.59 |
|  | C27 | 65 | F | Adenocarcinoma | Stage Ⅰ | 1.08 |
|  | C28 | 52 | F | Adenocarcinoma | Stage Ⅰ | 1.61 |
|  | C29 | 75 | M | Adenocarcinoma | Stage Ⅱ B | 5.64 |
|  | C30 | 68 | M | Adenocarcinoma | Stage Ⅱ A | 3.94 |
|  | C31 | 80 | M | Adenocarcinoma | Stage Ⅱ A | 3.35 |
|  | C32 | 47 | M | Adenocarcinoma | Stage Ⅱ A | 2.45 |
|  | C33 | 34 | M | Adenocarcinoma | Stage Ⅲ B | 2.55 |
|  | C34 | 54 | F | Adenocarcinoma | Stage Ⅲ B | 3.72 |
|  | C35 | 36 | M | Adenocarcinoma | Stage Ⅲ B | 1.44 |
|  | C36 | 63 | F | Adenocarcinoma | Stage Ⅲ B | 1.15 |
|  | C37 | 53 | F | Adenocarcinoma | Stage Ⅳ | 187 |
|  | C38 | 67 | M | Adenocarcinoma | Stage Ⅳ | 5.64 |
|  | C39 | 72 | F | Adenocarcinoma | Stage Ⅳ | 1.03 |
| **Benign**  **colorectal tumor** | B1 | 73 | F | Tubulovillous adenoma with focal high grade dysplasia | - | - |
|  | B2 | 73 | F | Tubular adenoma |  | 4.13 |
|  | B3 | 53 | F | Tubulovillous adenoma with focal high grade dysplasia |  | 0.3 |
|  | B4 | 68 | M | Traditional serrated adenoma |  | 2.11 |
|  | B5 | 64 | F | Tubulovillous adenoma with focal high grade dysplasia |  | 3.94 |
|  | B6 | 48 | F | Tubulovillous adenoma with low grade dysplasia |  | - |
|  | B7 | 60 | M | Tubular adenoma with high grade dysplasia |  | 0.59 |
|  | B8 | 55 | F | Tubular adenoma with low grade dysplasia |  | - |
|  | B9 | 58 | F | Tubulovillous adenoma with focal high grade dysplasia |  | - |
|  | B10 | 72 | F | Tubular adenoma with focal high grade dysplasia |  | - |
|  | B11 | 66 | F | Tubulovillous adenoma with low grade dysplasia |  | - |
|  | B12 | 66 | M | Tubular adenoma with low grade dysplasia |  | 2.09 |
|  | B13 | 58 | M | Tubulovillous adenoma with focal high grade dysplasia |  | 1.05 |
|  | B14 | 66 | M | Tubular adenoma with low grade dysplasia |  | 2.09 |
|  | B15 | 68 | M | Tubular adenoma |  | 1.5 |
|  | B16 | 52 | M | Tubular adenoma with low grade dysplasia |  | - |
|  | B17 | 66 | F | Tubular adenoma with focal high grade dysplasia |  | - |
|  | B18 | 75 | M | Tubulovillous adenoma with focal high grade dysplasia |  | - |
|  | B19 | 64 | M | Tubulovillous adenoma with high grade dysplasia |  | 3.15 |

**Table S2. Tissue KRAS mutation detection by PNAClamp™ and sanger sequencing.** Red text indicates results that are inconsistent with the tissue KRAS mutation status.

| **Classification** | **Patient number** | **Tissue** | | **Plasma** | **Plasma** |
| --- | --- | --- | --- | --- | --- |
|  |  | **PNAClamp™ KRAS Mutation Detection Kit** | **Sanger sequencing** | **PNAClamp™ KRAS Mutation Detection Kit** | **ADPS™ KRAS Mutation Test Kit** |
| **Malignant colorectal tumor** | C1 | Codon 12 | p.G12V (c.35G>T) | Not detected | G12X |
|  | C2 | Codon 12 | p.G12V (c.35G>T) | Not detected | G12X |
|  | C3 | Codon 12 | p.G12D (c.35G>A) | Not detected | G12X |
|  | C4 | Codon 13 | p.G13D (c.38G>A) | Not detected | G12X  (misclassification) |
|  | C5 | Codon 13 | p.G13D  (c.38G>A) | Not detected | Not detected |
|  | C6 | Codon 12 | p.G12D (c.35G>A) | Not detected | G12X |
|  | C7 | Codon 12 | p.G12D (c.35G>A) | Not detected | Not detected |
|  | C8 | Codon 12 | p.G12D (c.35G>A) | Not detected | Not detected |
|  | C9 | Codon 13 | p.G13D (c.38G>A) | Not detected | G12X  (misclassification) |
|  | C10 | Codon 12 | p.G12V (c.35G>T) | Not detected | G12X |
|  | C11 | Codon 12 | p.G12D (c.35G>A) | Not detected | G12X |
|  | C12 | Codon 12 | p.G12D (c.35G>A) | Not detected | G12X |
|  | C13 | Codon 12 | p.G12D (c.35G>A) | Not detected | Not detected |
|  | C14 | Codon 12 | p.G12A  (c.35G>C) | Not detected | G12X |
|  | C15 | Codon 12 | p.G12D (c.35G>A) | Not detected | G12X |
|  | C16 | Codon 12 | p.G12D (c.35G>A) | Not detected | G12X |
|  | C17 | Codon 12  Codon 146 | p.G12A  (c.35G>C)  p.A146T  (c.436G>A) | Not detected | G12X |
|  | C18 | Codon 146 | p.A146V  (c.437C>T) | Not detected | Not detected |
|  | C19 | Wild | not analyzed | not analyzed | Wild |
|  | C20 | Wild |  |  | Wild |
|  | C21 | Wild |  |  | G12X  (False positive) |
|  | C22 | Wild |  |  | G12X  (False positive) |
|  | C23 | Wild |  |  | G12X  (False positive) |
|  | C24 | Wild |  |  | Wild |
|  | C25 | Wild |  |  | G12X  (False positive) |
|  | C26 | Wild |  |  | Wild |
|  | C27 | Wild |  |  | G12X  (False positive) |
|  | C28 | Wild |  |  | G12X  (False positive) |
|  | C29 | Wild |  |  | G12X  (False positive) |
|  | C30 | Wild |  |  | G12X  (False positive) |
|  | C31 | Wild |  |  | G12X  (False positive) |
|  | C32 | Wild |  |  | G12X  (False positive) |
|  | C33 | Wild |  |  | Wild |
|  | C34 | Wild |  |  | Wild |
|  | C35 | Wild |  |  | Wild |
|  | C36 | Wild |  |  | Wild |
|  | C37 | Wild |  |  | Wild |
|  | C38 | Wild |  |  | Wild |
|  | C39 | Wild |  |  | Wild |
| **Benign**  **colorectal tumor** | B1 | Codon 12 | p.G12D (c.35G>A) | Not analyzed | Not analyzed |
|  | B2 | Codon 12 | p.G12V (c.35G>T) |  |  |
|  | B3 | Codon 12 | p.G12D (c.35G>A) |  |  |
|  | B4 | Codon 12 | p.G12V (c.35G>T) |  |  |
|  | B5 | Codon 13 | p.G13D  (c.38G>A) |  |  |
|  | B6 | Codon 12 | p.G12D (c.35G>A) |  |  |
|  | B7 | Codon 13 | p.G13D  (c.38G>A) |  |  |
|  | B8 | Codon 12 | p.G12V (c.35G>T) |  |  |
|  | B9 | Codon 13 | p.G13D  (c.38G>A) |  |  |
|  | B10 | Codon 12 | p.G12D (c.35G>A) |  |  |
|  | B11 | Codon 13 | p.G13D  (c.38G>A) |  |  |
|  | B12 | Codon 61 | p.Q61H  (c.183A>C) |  |  |
|  | B13 | Codon 146 | p.A146T (c.436G>A) |  |  |
|  | B14 | Wild | - |  |  |
|  | B15 | Wild |  |  |  |
|  | B16 | Wild |  |  |  |
|  | B17 | Wild |  |  |  |
|  | B18 | Wild |  |  |  |
|  | B19 | Wild |  |  |  |

**Table S3. Comparative detection of KRAS mutations in colorectal cancer by PNAClamp™ and 3D nanoplasmonic KRAS mutation detection microarray.** Results highlighted in blue indicate concordance with the tissue KRAS mutation status, while results in red indicate discrepancies.

| **Classification** | **Patient number** | **PNAClamp™ KRAS Mutation Detection Kit** | **3D Nanoplasmonic**  **KRAS Mutation Detection**  **Microarray** | | |
| --- | --- | --- | --- | --- | --- |
|  |  | **Tissue** | **Tissue** | **Plasma** | **Urine** |
| **Malignant colorectal tumor** | C1 | Codon 12 | Codon 12/13 | Codon 12/13 | Codon 12/13 |
|  | C2 | Codon 12 | Codon 12/13 | Codon 12/13 | Codon 12/13 |
|  | C3 | Codon 12 | Codon 12/13 | Codon 12/13 | Codon 12/13 |
|  | C4 | Codon 13 | Codon 12/13 | Codon 12/13 | Codon 12/13 |
|  | C5 | Codon 13 | Codon 12/13 | Codon 12/13 | Codon 12/13 |
|  | C6 | Codon 12 | Codon 12/13 | Codon 12/13 | Codon 12/13 |
|  | C7 | Codon 12 | Codon 12/13 | Codon 12/13 | Codon 12/13 |
|  | C8 | Codon 12 | Codon 12/13 | Codon 12/13 | Codon 12/13 |
|  | C9 | Codon 13 | Codon 12/13 | Codon 12/13 | Codon 12/13 |
|  | C10 | Codon 12 | Codon 12/13 | Codon 12/13 | Codon 12/13 |
|  | C11 | Codon 12 | Codon 12/13 | Codon 12/13 | Codon 12/13 |
|  | C12 | Codon 12 | Codon 12/13 | Codon 12/13 | Codon 12/13 |
|  | C13 | Codon 12 | Codon 12/13 | Codon 12/13 | Codon 12/13 |
|  | C14 | Codon 12 | Codon 12/13 | Codon 12/13 | Codon 12/13 |
|  | C15 | Codon 12 | Codon 12/13 | Codon 12/13 | Codon 12/13 |
|  | C16 | Codon 12 | Codon 12/13 | Codon 12/13 | Codon 12/13 |
|  | C17 | Codon 12  Codon 146 | Codon 12/13  Codon 146 | Codon 12/13  Codon 146 | Codon 12/13  Codon 146 |
|  | C18 | Codon 146 | Codon 146 | Codon 146 | Codon 146 |
|  | C19 | Wild | Wild | Wild | Wild |
|  | C20 | Wild | Wild | Wild | Wild |
|  | C21 | Wild | Wild | Wild | Wild |
|  | C22 | Wild | Wild | Codon 12/13 | Wild |
|  | C23 | Wild | Wild | Wild | Wild |
|  | C24 | Wild | Wild | Wild | Wild |
|  | C25 | Wild | Wild | Wild | Wild |
|  | C26 | Wild | Wild | Wild | Wild |
|  | C27 | Wild | Wild | Wild | Wild |
|  | C28 | Wild | Wild | Wild | Wild |
|  | C29 | Wild | Wild | Wild | Wild |
|  | C30 | Wild | Wild | Wild | Wild |
|  | C31 | Wild | Wild | Wild | Wild |
|  | C32 | Wild | Wild | Codon 12/13 | Wild |
|  | C33 | Wild | Wild | Codon 12/13 | Wild |
|  | C34 | Wild | Wild | Wild | Wild |
|  | C35 | Wild | Wild | Wild | Wild |
|  | C36 | Wild | Wild | Wild | Wild |
|  | C37 | Wild | Wild | Wild | Wild |
|  | C38 | Wild | Wild | Wild | Wild |
|  | C39 | Wild | Wild | Wild | Wild |
| **Benign**  **colorectal tumor** | B1 | Codon 12 | Codon 12/13 | Wild | Wild |
|  | B2 | Codon 12 | Codon 12/13 | Wild | Wild |
|  | B3 | Codon 12 | Codon 12/13 | Wild | Wild |
|  | B4 | Codon 12 | Codon 12/13 | Wild | Wild |
|  | B5 | Codon 13 | Codon 12/13 | Wild | Wild |
|  | B6 | Codon 12 | Codon 12/13 | Wild | Wild |
|  | B7 | Codon 13 | Codon 12/13 | Wild | Wild |
|  | B8 | Codon 12 | Codon 12/13 | Wild | Wild |
|  | B9 | Codon 13 | Codon 12/13 | Wild | Wild |
|  | B10 | Codon 12 | Codon 12/13 | Wild | Wild |
|  | B11 | Codon 13 | Codon 12/13 | Wild | Codon 12/13 |
|  | B12 | Codon 61 | Codon 61 | Codon 61 | Wild |
|  | B13 | Codon 146 | Codon 146 | Codon 146 | Wild |
|  | B14 | Wild | Wild | Wild | Wild |
|  | B15 | Wild | Wild | Wild | Wild |
|  | B16 | Wild | Wild | Wild | Wild |
|  | B17 | Wild | Wild | Wild | Wild |
|  | B18 | Wild | Wild | Wild | Wild |
|  | B19 | Wild | Wild | Wild | Wild |
